# Nano-formulations in disease therapy: designs, advances, challenges, and future directions

**DOI:** 10.1186/s12951-025-03442-7

**Published:** 2025-05-30

**Authors:** YunYan Shi, Xiao Li, Zhiyuan Li, Jialin Sun, Tong Gao, Gang Wei, Qie Guo

**Affiliations:** 1 Department of Pharmacy, The Affiliated Hospital of Qingdao University, Qingdao, 266003 Shandong People’s Republic of China; 2 College of Chemistry and Chemical Engineering, Qingdao University, Qingdao, 266071 Shandong People’s Republic of China; 3 Medical Research Center, The Affiliated Hospital of Qingdao University, Qingdao, 266003 Shandong People’s Republic of China

**Keywords:** Nano-formulations, Design strategies, Disease therapy, Hybrid nanoparticle

## Abstract

Nano-formulations, as an innovative drug delivery system, offer distinct advantages in enhancing drug administration methods, improving bioavailability, promoting biodegradability, and enabling targeted delivery. By exploiting the unique size advantages of nano-formulations, therapeutic agents, including drugs, genes, and proteins, can be precisely reorganized at the microscale level. This modification not only facilitates the precise release of these agents but also significantly enhances their efficacy while minimizing adverse effects, thereby creating novel opportunities for treatment of a wide range of diseases. In this review, we discuss recent advancements, challenges, and future perspectives in nano-formulations for therapeutic applications. For this aim, we firstly introduce the development, design, synthesis, and action mechanisms of nano-formulations. Then, we summarize their applications in disease diagnosis and treatment, especially in fields of oncology, pulmonology, cardiology, endocrinology, dermatology, and ophthalmology. Furthermore, we address the challenges associated with the medical applications of nanomaterials, and provide an outlook on future directions based on these considerations. This review offers a comprehensive examination of the current applications and potential significance of nano-formulations in disease diagnosis and treatment, thereby contributing to the advancement of modern medical therapies.

**Graphical abstract:**

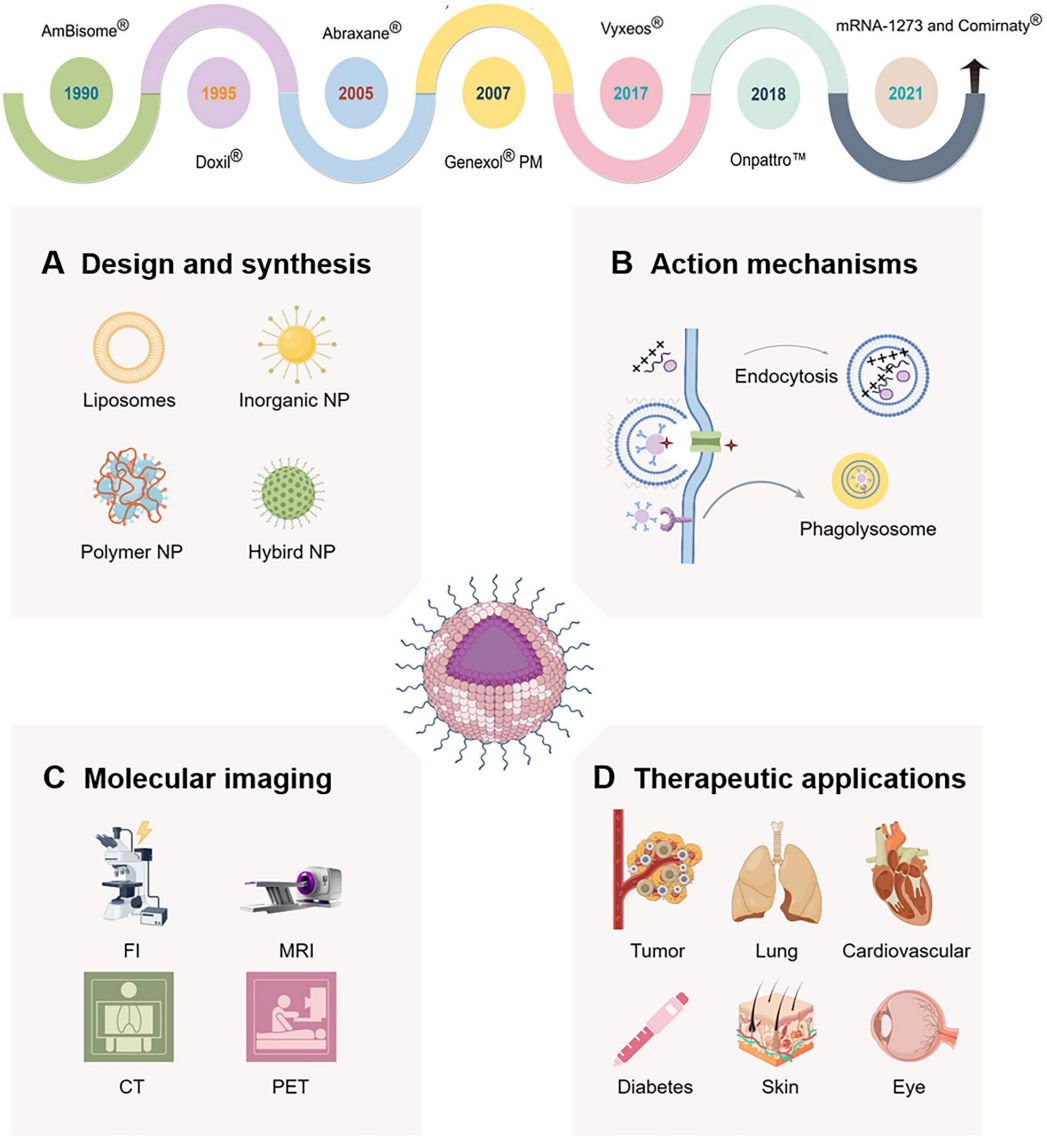

## Introduction

**Fig. 1:**
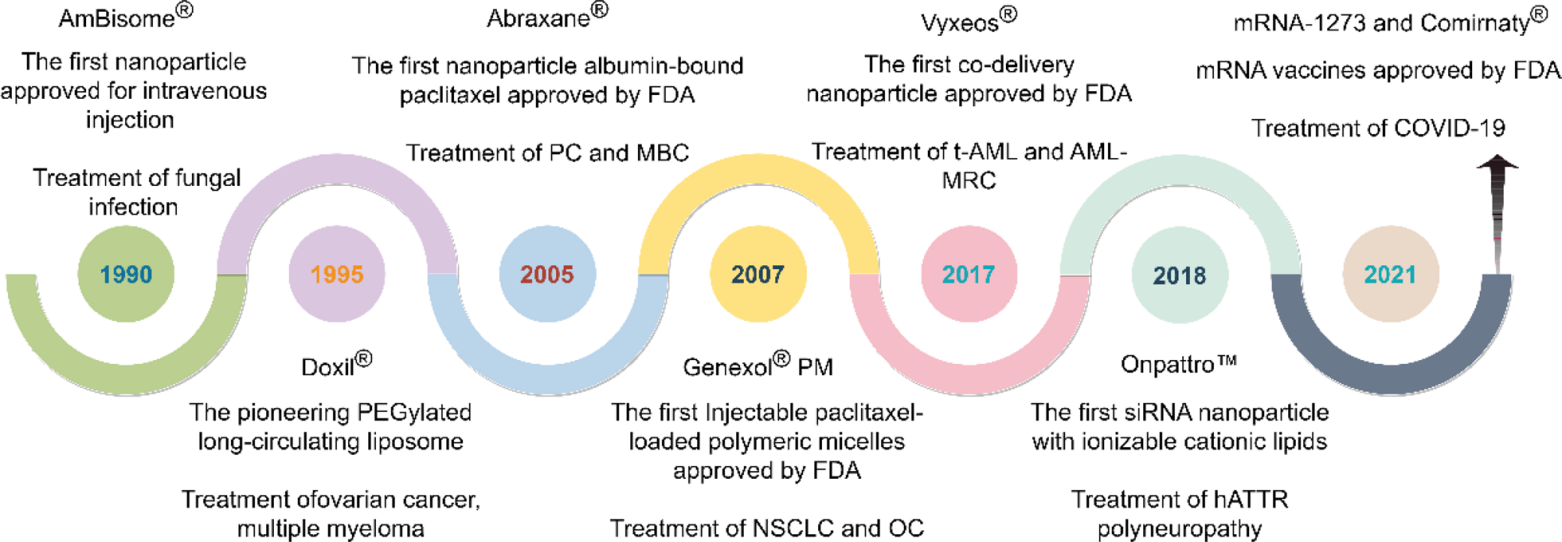
The historical development of nano-formulations (self-made image by authors)

Nano-formulations encompass drug particles at the nanoscale (1–1000 nm, preferably less than 500 nm) that are prepared using nanotechnology or developed as novel drug delivery systems by integrating drug particles with nano-formulations [1–3]. A brief history of nano-formulations within the fields of medicine and pharmacology dates to the 1960s (Fig. 1). In 1964, the structural characterization of liposomes was initially documented. Liposomes, vesicular structures formed by encapsulating drugs within phospholipid bilayers, are widely recognized for their excellent biocompatibility and biodegradability [4]. In 1971, Ryman et al*.* introduced the concept of employing liposomes as drug delivery vehicles, marking the beginning of research into nano-formulations [5].

In 1990, the first approved nano-formulation, Amphotericin B Liposome (trade name AmBisome^®^), was authorized for intravenous administration in Ireland to treat severe deep fungal infections, such as kala-azar, yeast infections, and coccidioidomycosis [6]. The pioneering PEGylated long-circulating liposomal drug, Doxil®, developed by Sequus Pharmaceuticals in the United States, received FDA approval for marketing in 1995, primarily indicated for advanced ovarian cancer, multiple myeloma, and HIV-associated Kaposi's sarcoma [7]. The first nanoparticle (NP) albumin-bound paclitaxel (Abraxane^®^) was approved by the FDA for marketing in 2005. It is indicated for the treatment of metastatic breast cancer (MBC) and pancreatic cancer (PC) [8]. Injectable paclitaxel-loaded polymeric micelles (Genexol^®^ PM), which received marketing approval in South Korea in 2007, represent the first polymeric nano-formulation approved for cancer therapy. Indications for Genexol^®^ PM include malignant tumors such as non-small cell lung cancer (NSCLC) and ovarian cancer (OC) [9]. In 2017, Vyxeos^®^, a liposomal formulation that concurrently encapsulates cytarabine and daunorubicin, was approved for commercial use in the treatment of conditions such as therapy-related acute myeloid leukemia (T-AML) and AML with myelodysplasia-related changes (AML-MRC) [10]. In 2018, the groundbreaking small interfering RNA (siRNA) drug Onpattro™ was introduced to the market, specifically for the treatment of hATTR polyneuropathy [11]. In the same year, Caplacizumab, the world's first nanobody drug, was approved for the treatment of adult acquired thrombotic thrombocytopenic purpura (aTTP). This event heralded the inception of a novel era in the advancement of pharmaceutical nano-formulations [12]. Then, in 2021, messenger RNA (mRNA) vaccines, specifically mRNA-1273 and BNT162b2 (commercially known as Comirnaty^®^), were authorized for emergency use in combating COVID-19 [13].

In recent years, a growing number of successful nano-formulations have emerged, including representative drugs that have either received FDA approval for marketing or are currently in clinical trial stages. Merck's Emend™ (aprepitant), developed using nanocrystal technology, represents the first and only Neurokinin-1 (NK-1) receptor antagonist on the market [14]. Insmed Incorporated’s Arikayce^®^ employs liposome-encapsulated amikacin to enhance pulmonary drug concentration while mitigating systemic toxicity [15]. Pfizer’s Besponsa^®^ conjugates an anti-CD22 antibody with a cytotoxic drug to deliver targeted therapy against B-cell acute lymphoblastic leukemia cells [16].

**Fig. 2:**
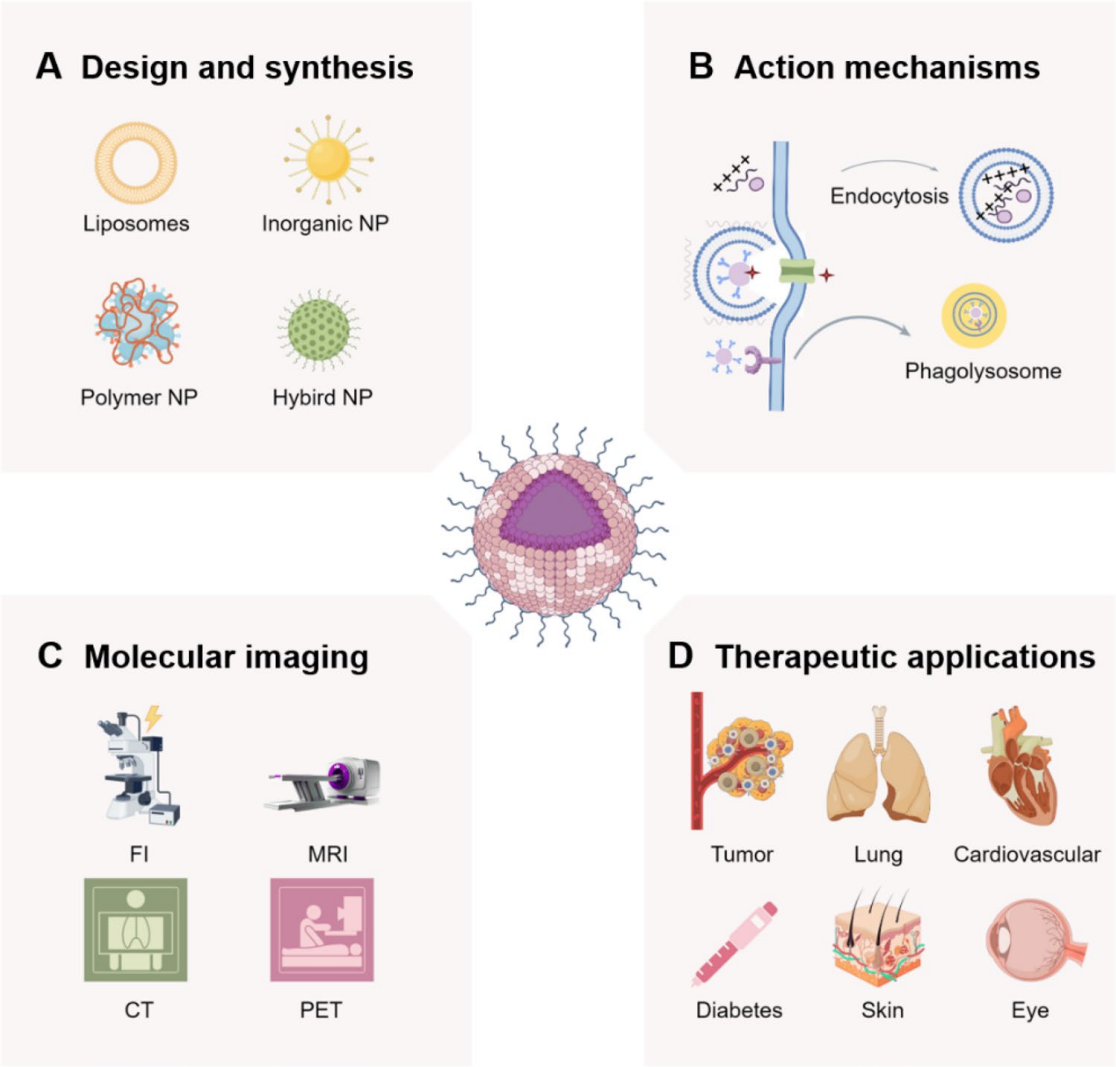
The application of nano-formulations in treating diverse diseases (self-made image by authors). **A** Design and synthesis. Synthesized nano-formulations can be categorized into four main types: liposomes, inorganic NP, polymer-based NP, and hybrid NP. **B** Action mechanisms. Action mechanisms of NPs primarily including cellular internalization, cytosolic delivery achieved through endosomal escape, and direct intracellular. **C** Molecular imaging. Nano-formulations play a pivotal role in disease diagnosis, encompassing modalities such as fluorescence imaging (FI), magnetic resonance imaging (MRI), computed tomography (CT), and positron emission tomography (PET). **D** Therapeutic applications. Nano-formulations find extensive applications in disease treatment across various medical specialties, including oncology, pulmonology, cardiology, endocrinology, dermatology, and ophthalmology

Over the past decade, the advancement of nano-formulations has evolved from fundamental materials science to clinical applications. FDA-approved nano-formulations (such as Doxil^®^, mRNA-LNP vaccines, etc.) have exhibited significant clinical efficacy. Concurrently, emerging technologies like stimulus-responsive nanocarriers, bionic nanoparticles, and multi-functional diagnostic-therapeutic integration platforms have propelled the evolution of personalized medicine. Notably, the incorporation of artificial intelligence-assisted (AI) design has established a novel paradigm for enhancing the precision and intelligence of nano-formulations. In particular, nano-formulations have been widely applied in modern medicine due to their unique physicochemical and biological properties, playing a significant role in various fields, including oncology, pulmonology, cardiology, endocrinology, dermatology, and ophthalmology (Fig. 2). However, the application of nano-formulations continues to encounter a multitude of challenges, such as quality control during large-scale production and long-term evaluations in vivo. This review provides an overview of the breakthroughs achieved in the application of nano-formulations for targeted delivery and disease diagnosis and treatment. It further examines the critical scientific issues bridging material innovation and clinical translation, while also exploring potential future development directions. Here, we elaborate on the application of novel nano-formulations in disease diagnosis and treatment based on disease classification, while analyzing the difficulties and challenges encountered during implementation. Such detailed and comprehensive research has been rigorously validated but remains rarely reported in the literature.

## Design and synthesis of nano-formulations

**Fig. 3:**
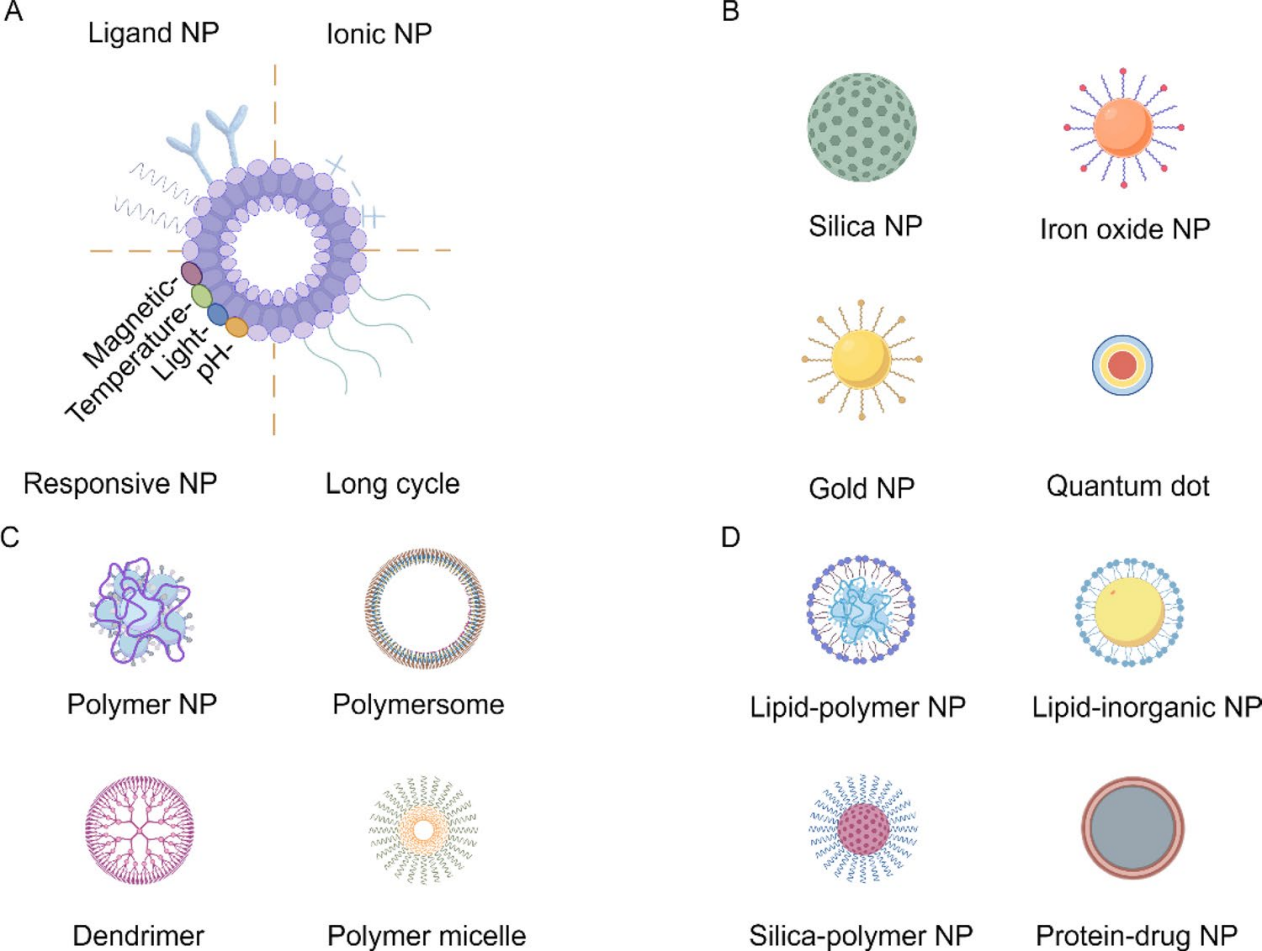
Different classes of nano-formulations. **A** liposomes: including ligand NP, ionic NP, responsive NP (containing magnetic NP, temperature NP, light NP, and pH NP), and long cycle NP; **B** inorganic nano-formulations: including silica NP, iron oxide NP, gold NP, and Quantum dot; **C** polymer nano-formulations: including polymer NP, polymersome NP, dendrimer, and polymer micelle; **D** hybrid nano-formulations: including lipid-polymer NP, lipid-inorganic NP, silica-polymer NP, and protein-drug NP

Currently, nano-formulations used in pharmaceutical nano-formulations can be classified into liposomes, inorganic nano-formulations, polymer-based nano-formulations, and hybrid nano-formulations (Fig. 3). Various types of nano-formulations possess unique characteristics. For instance, liposomes, as vesicular nano-formulations, exhibit excellent biocompatibility and can enhance the stability, bioavailability, and distribution of drugs. In pharmaceutical formulations, liposomes represent a relatively mature and widely used nanocarrier technology. Inorganic nano-formulations as delivery systems primarily composed of inorganic substances and have garnered significant attention due to their unique electrical and optical properties, biocompatibility, and low cytotoxicity. Polymeric NPs consist of polymeric materials, including natural polymers, such as albumin and polysaccharides, or synthetic polymers like polylactic acid (PLA) and polyglycolic acid (PGA). Drugs are dissolved, encapsulated, or adsorbed within the polymeric NP matrix, essentially forming a homogenous system. Hybrid nano-formulations combine multiple materials or properties, aiming to improve drug delivery efficiency and therapeutic effectiveness [17, 18].

Compared with traditional drugs, nano-formulations significantly enhance their bioavailability. For example, the natural plant alkaloid Camptothecin (CPT) exhibits potent antitumor activity; however, its structural instability and insolubility limit its applications. CPT nano-formulations can effectively overcome these shortcomings [19]. Additionally, nano-formulations coated with synthetic polymers offer the advantage of shielding drugs from immune-mediated degradation [20]. Additionally, nano-formulations naturally possess a high surface area-to-volume ratio, and can be functionalized with various ligands, including receptors, or antigens, lactoferrin, and folic acid. These modifications improve their ability to cross biological barriers and actively target treatment sites, thereby enhancing therapeutic efficacy while reducing side effects [4, 21–24]. Moreover, multifunctional stimuli-responsive nano-systems, engineered from stimuli-responsive materials, can selectively release therapeutic agents in response to specific environmental triggers at the disease site, significantly enhancing drug accumulation in the target tissue [25]. Besides, diagnostic molecules such as fluorescent dyes, contrast agents, and biomarker detection probes can be conjugated to or integrated with nano-formulations, thereby enabling the development of an integrated nano-system for diagnosis, treatment, and monitoring [26].

In the past decade, a promising biomimetic targeting strategy has attracted significant attention [27, 28]. In the field of biomimetic membranes, cell membranes are predominantly sourced from various types of cells including cancer cells, neutrophils, leukocytes, natural killer (NK) cells, macrophages, red blood cells, or combinations thereof [29–32]. Besides intact cell membranes, extracellular vesicles (including exosomes, microvesicles, and apoptotic bodies) and various viral vectors originating from mammalian viruses, bacteriophages, and plant viruses also serve as natural membrane substrates [33, 34]. These vectors interact with synthetic NPs or drug-loaded functional agents to form biomimetic NPs (BNPs) via diverse methodologies [35]. BNPs minimize adverse immune responses and avoid direct elimination, thereby prolonging their circulation time in the body [36]. Dendritic cell membrane-coated NPs loaded with drugs have demonstrated efficacy in both immunotherapy and immunoprevention in mouse models [37]. For example, Fe_3_O_4_-ICG@IRM, encapsulated within a hybrid biomimetic membrane derived from mouse ID8 ovarian cancer cells and red blood cell membranes and incorporating indocyanine green, demonstrates potential for the treatment of ovarian cancer [32].

Optimizing the physicochemical properties of nano-formulations can balance long circulation, targeting and biocompatibility, but the design needs to be adjusted according to specific applications (such as tumor delivery, gene therapy, etc.).The size of nano-formulations affects the distribution and removal. Nano-preparations with small particle size (< 10 nm) are easily cleared by glomerular filtration and may also penetrate the vascular endothelial space [38]. The nano-formulations with medium size (10–200 nm) are beneficial for prolongating blood circulation to avoid liver and spleen entrapment, thereby targeting tumor tissue through the enhanced permeability and retention (EPR) effect [39]. Nano-formulations with large particle size (> 500 nm) are easy to be phagocytosed by the reticuloendothelial system (RES) and are enriched in the liver and spleen [40]. In addition, the nano-formulations with 20–200 nm are more likely to enter cells through endocytosis; the nano-formulations with such as < 10 nm may be passive diffusion, and too large nanoparticle cannot be effectively eliminated [41].

Positively charged nano-formulations can bind to negatively charged cell membranes, thereby enhancing endocytosis efficiency. However, they may also increase non-specific adsorption, cytotoxicity, and plasma protein adsorption, which could modulate physicochemical interactions [42, 43]. Thus, these nano-formulations can be expedited eliminated. The cycle time of negatively charged or neutral nano-formulations can be prolonged by minimizing electrostatic interactions with cell membranes. However, this may result in a weakened targeting effect [44]. Spherical nano-formulations are the most common type, characterized by ease of synthesis and high endocytosis efficiency [45]. Nano-formulations with a high aspect ratio (such as nanorods and fibers) have the potential to prolong blood circulation time; however, they are prone to being captured by the RES [46]. There is enhanced vascular wall adhesion or tumor-targeting in nano-formulations containing specifically shaped nano-formulations (e.g., disk-shaped). Irregularly shaped nano-formulations may elicit immune responses or cause mechanical damage [47].

In general, the combined effects of nano-formulations on biological interactions are multifaceted and complex. Pharmacokinetic factors, including particle size and surface charge, play critical roles in determining blood circulation time, tissue distribution, and clearance pathways. The EPR effects is dependent on an optimal range of particle size, while active targeting necessitates specific surface modifications, such as the conjugation of antibodies or ligands. Positively charged or excessively large nano-formulations may lead to inflammation or organ deposition, particularly in organs such as the liver and spleen. Additionally, the shape and charge of nanomaterials significantly influence the mechanisms of endocytosis (e.g. Clathrin vs. fossa pathway).

The design strategies for nano-formulations encompass targeted design, stimulus-responsive design, and multifunctional integration design. Targeted design includes (i) passive targeting, which capitalizes on the characteristics of the tumor microenvironment (such as increased vascular permeability and impaired lymphatic drainage) to enable nano-formulations to accumulate in tumor sites via the EPR effect; and (ii) active targeting, which involves modifying the surface of nanoparticles to enable them to specifically recognize and bind to target cells or tissues, thereby enhancing drug accumulation at the disease site [48]. In stimulus-responsive design, nano-formulations can release drugs in response to changes in pH, enzymes, or REDOX environments. Temperature-responsive systems utilize localized heating (such as through ultrasound or radiofrequency) to induce phase transitions or structural changes in the nanocarriers, leading to drug release. Additionally, ultrasound-sensitive nanosystems can be designed to trigger drug release through the radiative force or cavitation effects of ultrasound [49]. Multifunctional integration design integrates multiple therapeutic modalities (such as chemotherapy, radiotherapy, and immunotherapy) onto a single nanoscale platform to achieve synergistic therapeutic effects and enhance treatment efficacy. Furthermore, nano-formulations with both diagnostic (e.g., imaging) and therapeutic functionalities can be designed to enable theranostic applications [50].

The synthesis methods of nano-formulations encompass chemical synthesis, self-assembly, and solvent evaporation. Chemical synthesis methods include: (i) sol–gel method, which involves the hydrolysis and condensation reactions of metal alkoxides to form NPs; (ii) co-precipitation method, where multiple metal ions are simultaneously precipitated under controlled reaction conditions to form composite NPs; (iii) microemulsion method, utilizing surfactant-stabilized microemulsion droplets as nanoreactors for synthesizing NPs; and (iv) hydrothermal synthesis method, which employs aqueous solution-based chemical reactions under high temperature and pressure conditions to synthesize NPs [1, 51]. Molecular self-assembly is a method that spontaneously forms nanostructures through intermolecular interactions such as electrostatic attraction, hydrogen bonding, π-π stacking, etc. Additionally, NP self-assembly involves controlling the surface chemical properties of NPs to induce their spontaneous assembly into nanomaterials with specific structures and functions [52, 53]. The solvent evaporation method involves dissolving the drug in an organic phase (e.g., chloroform), emulsifying it with an aqueous phase, and then evaporating the organic solvent to form drug-loaded NPs [54]. In practical applications, it is necessary to select appropriate methods based on drug properties, nanomaterial types, and therapeutic requirements. For instance, the emulsion-solvent evaporation method is frequently employed for the nanonization of antibiotics, whereas the sol–gel method and hydrothermal synthesis are more suitable for the preparation of inorganic non-metallic nanomaterials [55].

## Action mechanisms, metabolism and excretion of nano-formulations

**Fig. 4:**
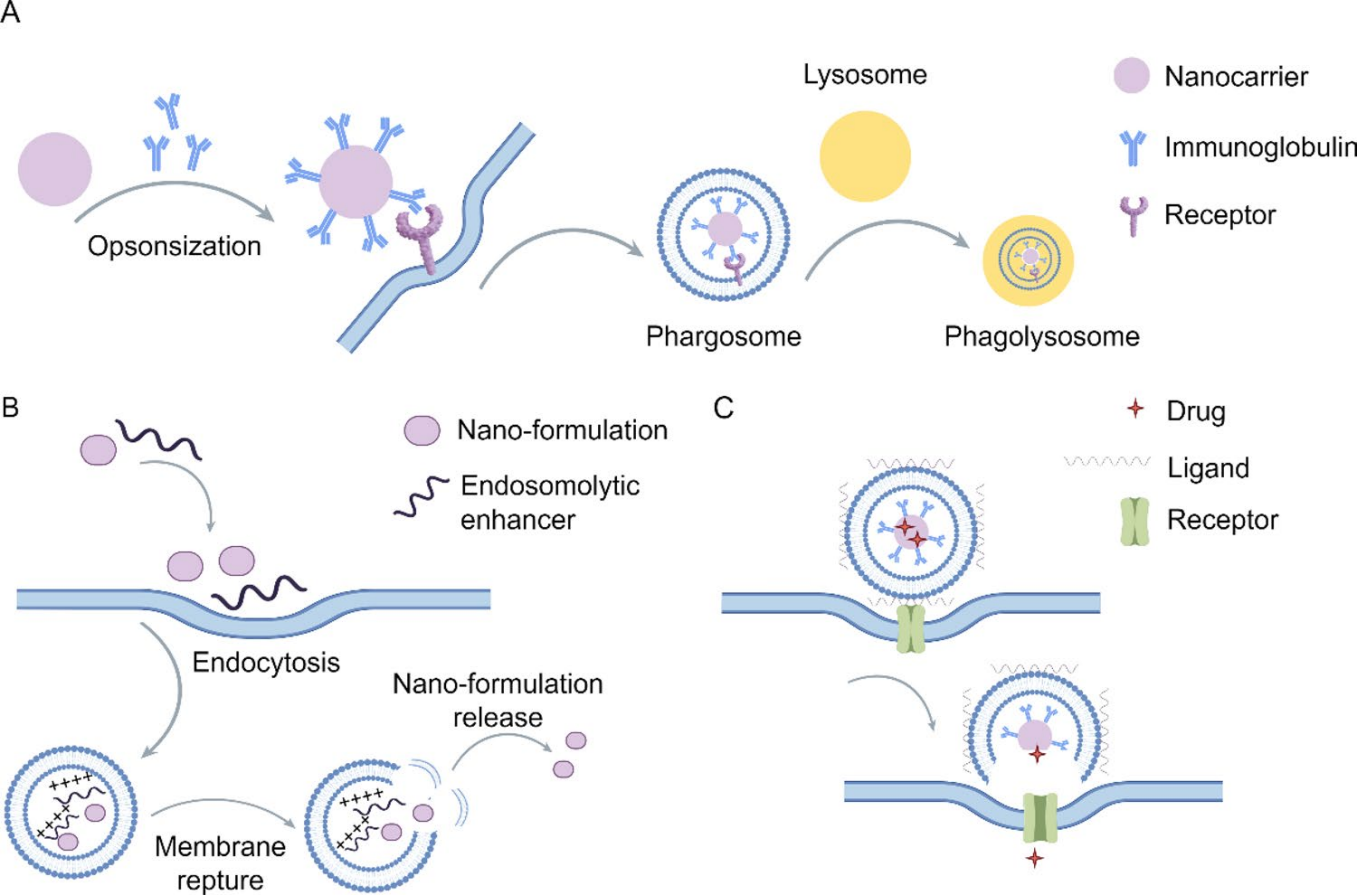
Major NP-based delivery methods: **A** cellular internalization, **B** cytosolic delivery through endosomal escape, and **C** direct intracellular delivery

Drug delivery via NPs primarily involves three approaches, including cellular internalization, cytosolic delivery achieved through endosomal escape, and direct intracellular, as presented in Fig. 4.

Nano-formulations, by binding ligands on their surfaces to specific receptors or other molecules onto the cell membrane, are internalized by cells through endocytosis and subsequently degraded via the endosome-lysosome transport pathway (Fig. 4A). The lysosome is a critical cellular organelle that houses a diverse array of hydrolytic enzymes capable of degrading materials derived from both intracellular and extracellular sources. Nano-formulations are degraded by hydrolytic enzymes within the lysosome, leading to the loss of their original structure and function and the subsequent release of the encapsulated drug. However, NPs engineered with specific properties, such as modifications in size, shape, or surface chemistry, can evade degradation within the endosome/lysosome compartment and successfully translocate into the cytoplasm. This phenomenon is referred to as endosomal/lysosomal escape (Fig. 4B). Once the NPs have escaped from the endosome and entered the cytoplasm, they can begin to release the drug. The mechanism of drug release can be diffusion-controlled, erosion-controlled, or stimulus-responsive, depending on factors such as the composition, structure, and method of drug loading.

In fact, nano-formulations can not only improve the solubility, pharmacokinetics and drug retention time of poorly soluble drugs but also enable controlled drug release through the stimulus-responsive properties of the carrier [56–58]. Direct intracellular delivery methods generally employ physical or chemical approaches to introduce NPs or their associated payloads directly into the cell interior (Fig. 4C). These methods encompass microinjection, direct intracellular delivery, and membrane permeabilization. Moreover, its nanoscale dimensions enable more effective interaction with the biological environment. The EPR effect facilitates the migration of macromolecules up to 400 nm in diameter to the tumor site, thereby enabling passive targeting of the nano-preparations to the tumor cell [21, 59]. Since its discovery in 1986, the EPR effect has served as a fundamental pillar in the advancement of cancer nano-therapy [60].

The metabolism and excretion of nano-formulations are the key factors that significantly influence their efficacy and safety. Owing to the distinctive physical and chemical characteristics of nano-formulations (e.g., small size, high surface area, modifiable surface functionality, etc.), the metabolic and excretory pathways of various nano-formulations markedly differ from those of conventional small-molecule drugs. Nano-formulations are typically metabolized through enzymatic degradation, redox reactions, and protein corona formation. In particular, some nano-formulations (e.g., liposomes, polymer nanoparticles) can be degraded into small molecule products by enzymes in the body (e.g., esterases, proteases) [61]. Metal-containing nanoparticles (e.g., gold, iron oxides) may be modified or decomposed through REDOX reactions. After the nanoparticles enter the blood, the surface will adsorb proteins to form a “protein crown”, which may change their original properties and affect metabolic pathways [62, 63]. It should be noted that many nanomaterials (such as inorganic nanoparticles) metabolize slowly and may remain in the body for a long time in their original form.

Excretion of nano-formulations primarily depends on their size, material composition, and surface characteristics. Typically, nanoparticles with a hydrated diameter of less than 5–6 nm can be filtered by the glomerulus and subsequently excreted via urine [64]. Larger particles or rigid structures, such as carbon nanotubes, are challenging to excrete via renal filtration due to their size and structural properties. Nanoparticles with larger dimensions (e.g., 20–200 nm) are more likely to be internalized by liver Kupffer cells or hepatocytes, subsequently entering the intestine through bile secretion and being excreted in feces [65, 66]. Particles with hydrophobic or negatively charged surfaces are more likely to be captured by the liver [38]. NPs are easily captured by mononuclear phagocytic system (MPS) such as liver, spleen, bone marrow) and remain for a long time, which may take months to slowly degrade and be excreted [67].

There are also notable challenges associated with the metabolism and excretion of nano-formulations. First, non-degradable nano-formulations may accumulate in the liver, spleen and other organs for a long time, and the potential toxicity needs to be evaluated [68]. Second, certain nano-formulations may activate the complement system or trigger inflammation [69]. In addition, patients with abnormal liver and kidney function may affect the excretion efficiency, and the dose needs to be adjusted [70]. Currently, imaging tracing techniques such as radiolabelling (e.g.^99m^Tc), fluorescent labelling or mass spectrometry are used to monitor the distribution of nano-formulations in vivo in real time [71–73]. Metabolomics can also be used to analyze the effects of nano-formulation on endogenous metabolic pathways [74].

In summary, the metabolic and excretory processes of nano-formulations are highly influenced by their design parameters, including size, material composition, and surface modification. Fine-tuning these parameters can modulate their behavior in vivo, thereby achieving an optimal balance between efficacy and safety. Future research should prioritize investigating long-term retention effects and developing precise strategies for enhancing excretion.

## Nano-formulations in treating cancers

Cancer poses one of the most significant public health challenges globally, with projections indicating approximately 30 million annual deaths from the disease by 2030 [75]. Traditional cancer treatments, including surgery, chemotherapy, and radiotherapy, have notably enhanced the survival rates of numerous patients. However, in the context of advanced metastatic cancer, their therapeutic efficacy is limited [76]. For many cancers, chemotherapy serves as the primary treatment option [77]. Nevertheless, conventional chemotherapy drugs are non-selective, causing indiscriminate damage to both tumor cells and normal cells, which often leads to toxic and adverse effects alongside therapeutic benefits. Furthermore, the emergence of multidrug resistance to these chemotherapy agents poses another significant challenge that cannot be overlooked [78]. Prolonging the survival and quality of life of patients by reducing the systemic toxicity of chemotherapy is the main goal of cancer treatment [79]. Therefore, investigating innovative therapeutic strategies, particularly nano-formulations that are both highly efficient and biologically safe, has emerged as a critical focus in cancer treatment.

### Nano-formulations and cancer diagnosis

**Fig. 5:**
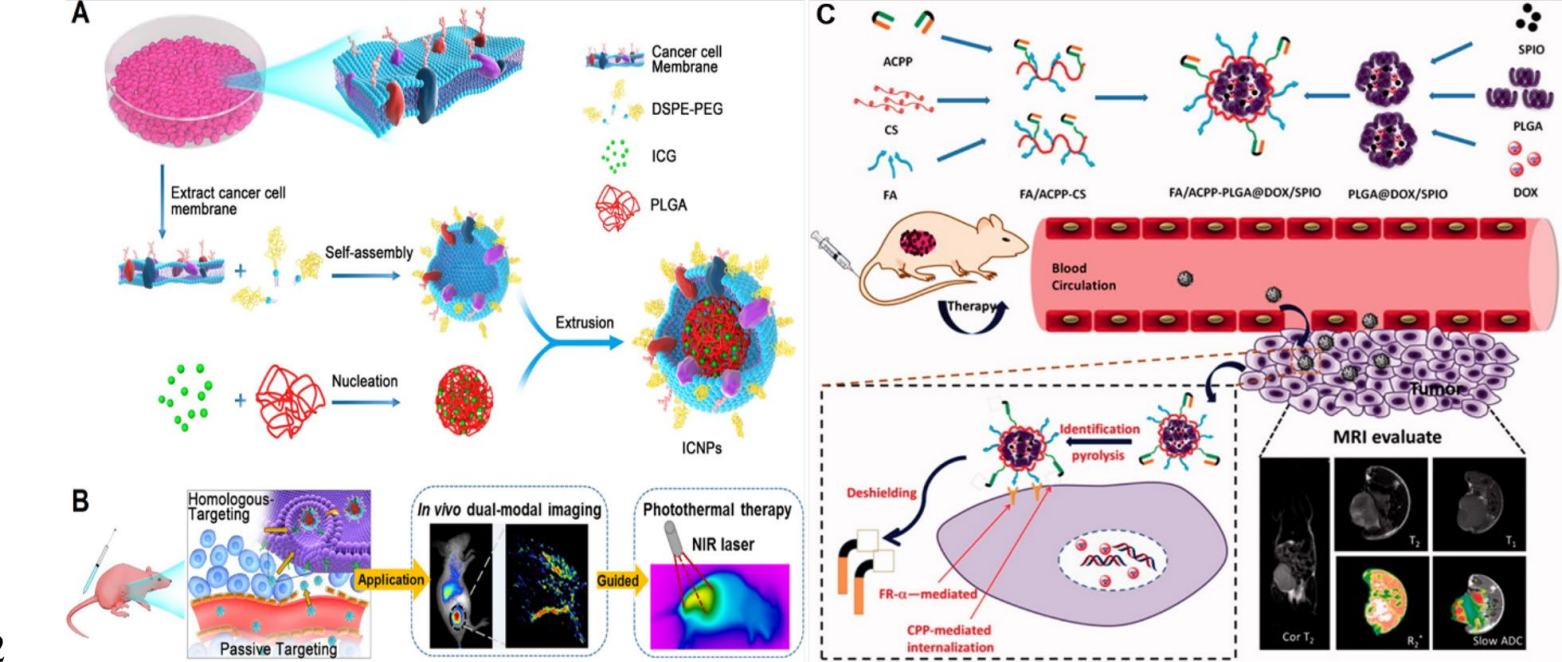
Schematic model of nano-formulations and cancer diagnosis techniques. **A** Preparation process of indocyanine green (ICG)-loaded and cancer cell membrane-coated nanoparticles (ICNPs). **B** Schematic diagram illustrating isotope-targeted ICNPs utilized for dual-modal imaging-guided photothermal therapy. **C** Synthetic design of F/A-PLGA@DOX/SPIO nanoparticles and schematic representation of their application in tumor MRI *Adapted from Chen Z, Zhao P, Luo Z, *et al*. Cancer Cell Membrane-Biomimetic Nanoparticles for Homologous-Targeting Dual-Modal Imaging and Photothermal Therapy. ACS Nano. 2016;10(11):10,049–57. Gao P, Mei C, He L, *et al*. Designing multifunctional cancer-targeted nanosystem for magnetic resonance molecular imaging-guided theranostics of lung cancer. Drug Deliv. 2018;25(1):1811–25.*

Early detection of cancer is essential for improving treatment outcomes and patient survival rates [80]. NPs have emerged as promising tools for the targeted delivery of diagnostic and chemotherapeutic agents to cancer cells. Nanodiscs (NDs), which are structurally analogous to high-density lipoproteins (HDLs), consist of nanoscale membrane bilayers encircled by amphiphilic molecules, including proteins, synthetic polymers, or short-chain lipids [81]. NDs with a nanoscale, disc-like morphology are readily amenable to conjugation with a variety of imaging agents [82]. Cancer cells exhibit elevated expression levels of specific receptors, including Scavenger Receptor Class B Type 1 (SR-B1), αvβ3 integrins, somatostatin receptors, folate receptors (FARs), and low-density lipoprotein (LDL) receptors [83]. NDs functionalized with specific tumor-targeting ligands chemically attached to their surface or edges can selectively target tumor cells, and have emerged as crucial tools for both diagnosis and treatment, as summarized in Fig. 5.

Currently, a wealth of evidence demonstrates the efficacy of NDs in delivering diagnostic reagents across various imaging modalities, including fluorescence imaging (FI), magnetic resonance imaging (MRI), computed tomography (CT), and positron emission tomography (PET) [84]. SR-B1 is highly expressed in various cancers, including prostate cancer, breast cancer, and ovarian cancer [85]. Tang et al*.* developed synthetic HDL (sHDL) and its pegylated counterpart (PEG-sHDL), while comparing their tumor targeting capabilities with NDs possessing liposomes and pegylated liposomes (PEG-LIP). By incorporating hydrophobic fluorescent dyes as model drugs and tracers, the cellular uptake efficiency, tumor spheroid penetration, tumor accumulation, and in vivo distribution of all NDs were systematically evaluated. The results demonstrated that sHDL significantly enhanced SR-BI-mediated tumor targeting, penetration of tumor tissues, and accumulation in tumors [86]. Chen and co-workers developed gadolinium and fluorescent dye-loaded, Arg-Gly-Asp (RGD)-modified reconstructed high-density lipoprotein (rHDL-RGD) NPs, which were further functionalized with a cyclic pentameric RGD peptide specific to αvβ3 integrin, for the targeted delivery to angiogenic endothelial cells. The in vitro studies demonstrated that rHDL-RGD NPs were selectively internalized by endothelial cells. Both near-infrared (NIR) and MR imaging confirmed the accumulation of rHDL-RGD in mouse tumor models, demonstrating the potential of NDs for multimodal imaging of tumor-associated processes [87].

Additionally, magnetic NPs (MNPs) exhibit superparamagnetism and exceptional biocompatibility, making them ideal carriers for immunodiagnostics. Specifically, iron oxide-based MNPs have garnered FDA approval for clinical applications as contrast agents in MRI [88]. Gao et al*.* have developed a multifunctional drug-loaded targeted nanosystem (F/A-PLGA@DOX/SPIO) that not only promotes excessive production of reactive oxygen species (ROS) in A549 lung cancer cells, inducing apoptosis, but also serves as an excellent T_2_-negative contrast agent for MRI [89]. Cancer cell membrane-coated NPs (CCM-NPs) exhibited intrinsic tumor-homing properties, rendering them exceptionally suitable for diagnostic imaging and targeted phototherapy in cancer treatment. In another case, Chen et al*.* designed ICNPs consisting of an indocyanine green (ICG) polymer core encapsulated within a cancer cell membrane shell, enabling specific homologous targeting of cancer cells. Under near-infrared (NIR) laser irradiation, ICNPs effectively eliminated xenograft tumors via photothermal therapy (PTT) and concurrently demonstrated superior fluorescence/photoacoustic (FL/PA) imaging capabilities [31].

### Nano-formulations and cancer metastasis

**Fig. 6:**
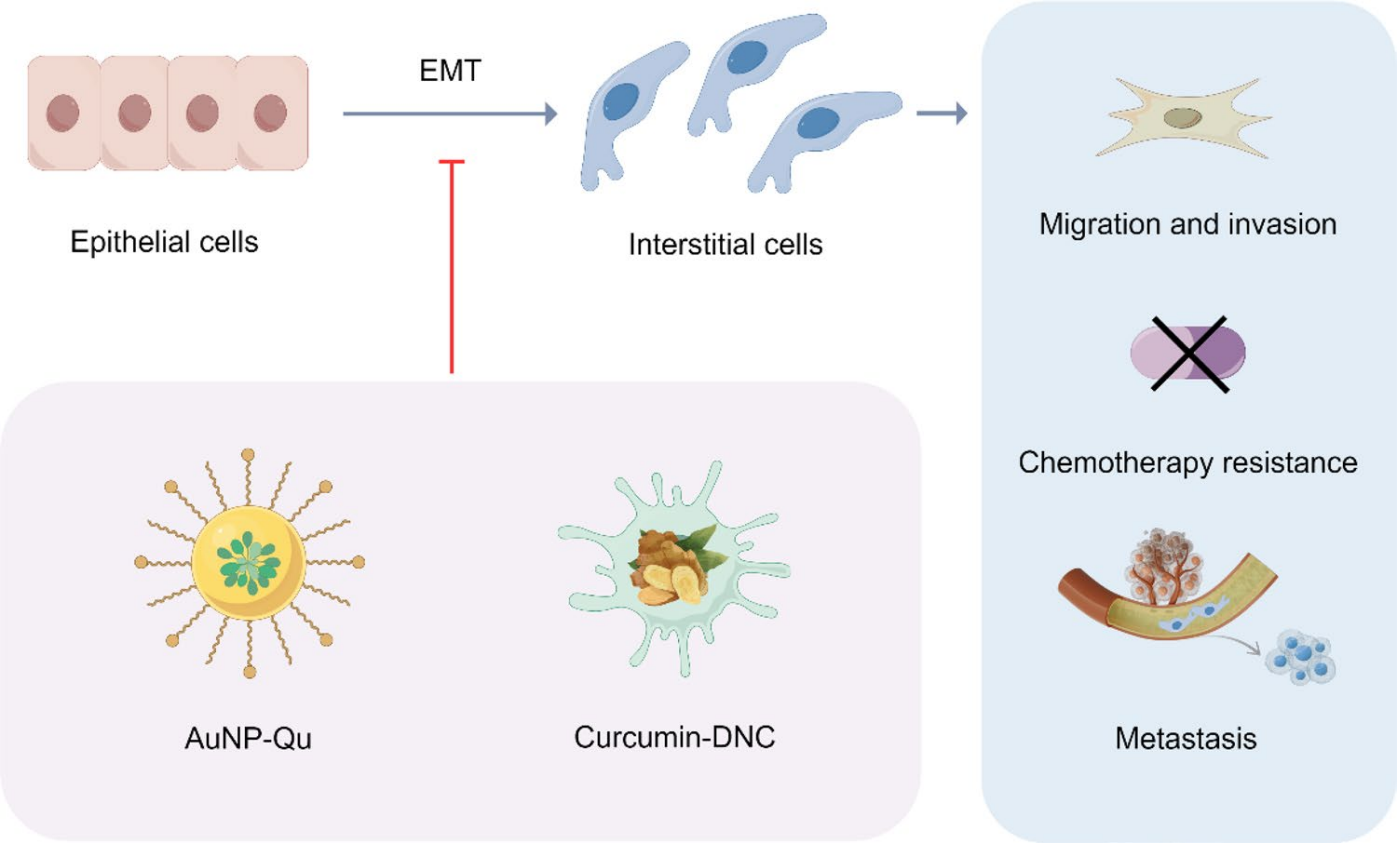
Schematic model of nano-formulations and cancer metastasis

Epithelial-to-Mesenchymal Transition (EMT) is a cellular process wherein epithelial cells lose their polarized organization and intercellular junctions, undergo morphological and cytoskeletal reorganization, and acquire mesenchymal traits, including fibroblast-like morphology, enhanced migratory capacity, and invasive capabilities [90]. Various cytokines present in the tumor microenvironment can activate a series of intracellular signaling pathways, inducing the occurrence of EMT [91]. The abnormal activation of EMT is not only essential for early tumor migration but also promotes chemotherapy resistance and disease progression [92]. Bioactive natural compounds, including artemisinin, curcumin, luteolin, withaferin-A (WFA), and quercetin, have been demonstrated to target the EMT signaling pathway and inhibit the expression of specific biomarkers in both cell cultures and preclinical cancer models [93]. However, the limited water solubility and low bioavailability of natural compounds significantly restrict their clinical applications. Recently, nano-formulations have emerged as a promising carrier for delivering these compounds, as demonstrated in Fig. 6.

The conjugate of gold NPs (AuNPs) and quercetin (AuNPs-Qu-5) demonstrates a significant capability to reduce the expression of proteins associated with EMT and angiogenesis, while concurrently enhancing the expression of E-cadherin. AuNPs-Qu-5 not only inhibited the migration and invasion of breast cancer cells but also suppressed the growth of breast cancer tumors in rats [94]. Agarwalla et al*.* have developed a co-formulation named GNP-DSH-WFA by conjugating thiol-modified dexamethasone (a glucocorticoid) and WFA onto gold NPs [95]. This formulation exhibited remarkable efficacy in a mouse melanoma model, effectively reversing EMT in tumor cells. Furthermore, GNP-DSH-WFA markedly decreased the expression of ATP-binding cassette subfamily G member 2 (ABCG2), a protein frequently observed in cancer cells and strongly linked to the development of multidrug resistance. By downregulating ABCG2 expression, GNP-DSH-WFA enhances the sensitivity of cancer cells to chemotherapeutic agents, thereby improving treatment efficacy [95].

Recently, Baghi et al*.* have demonstrated that the combination of curcumin-loaded dendrosomal nanocurcumin (DNC) with exogenously delivered p53 significantly reduced the transcription levels of ZEB1 and BMI1 genes, which were associated with EMT and metastasis [96]. DNC, in combination with exogenous p53, demonstrated synergistic anticancer effects by significantly enhancing apoptosis and reducing migratory capacity in breast cancer MDA-MB-231 cells. In another study, Manisha and co-workers designed selenium NPs loaded with curcumin (Se-CurNPs), which exerted anticancer effects in colorectal cancer cells (HCT116) by enhancing autophagy and apoptosis levels, while significantly reducing the expression of EMT-related metastasis proteins (such as CD44 and N-cadherin) [97]. Furthermore, Se-CurNPs demonstrated a significant reduction in tumor burden in tumor-bearing mice and extended their average survival time [97, 98]. Additionally, researchers have developed a bimetallic nano-formulation platform loaded with PtCl₂(OH)₂(NH₃)₂, utilizing pH-sensitive zeolite imidazolate framework-8 (ZIF-8) as a carrier. This nano-formulation inhibits the proliferation and invasion of tumor cells by interfering with inositol-1,4,5-trisphosphate-mediated cell communication [99].

### Nano-formulations for cancer therapy

**Fig. 7:**
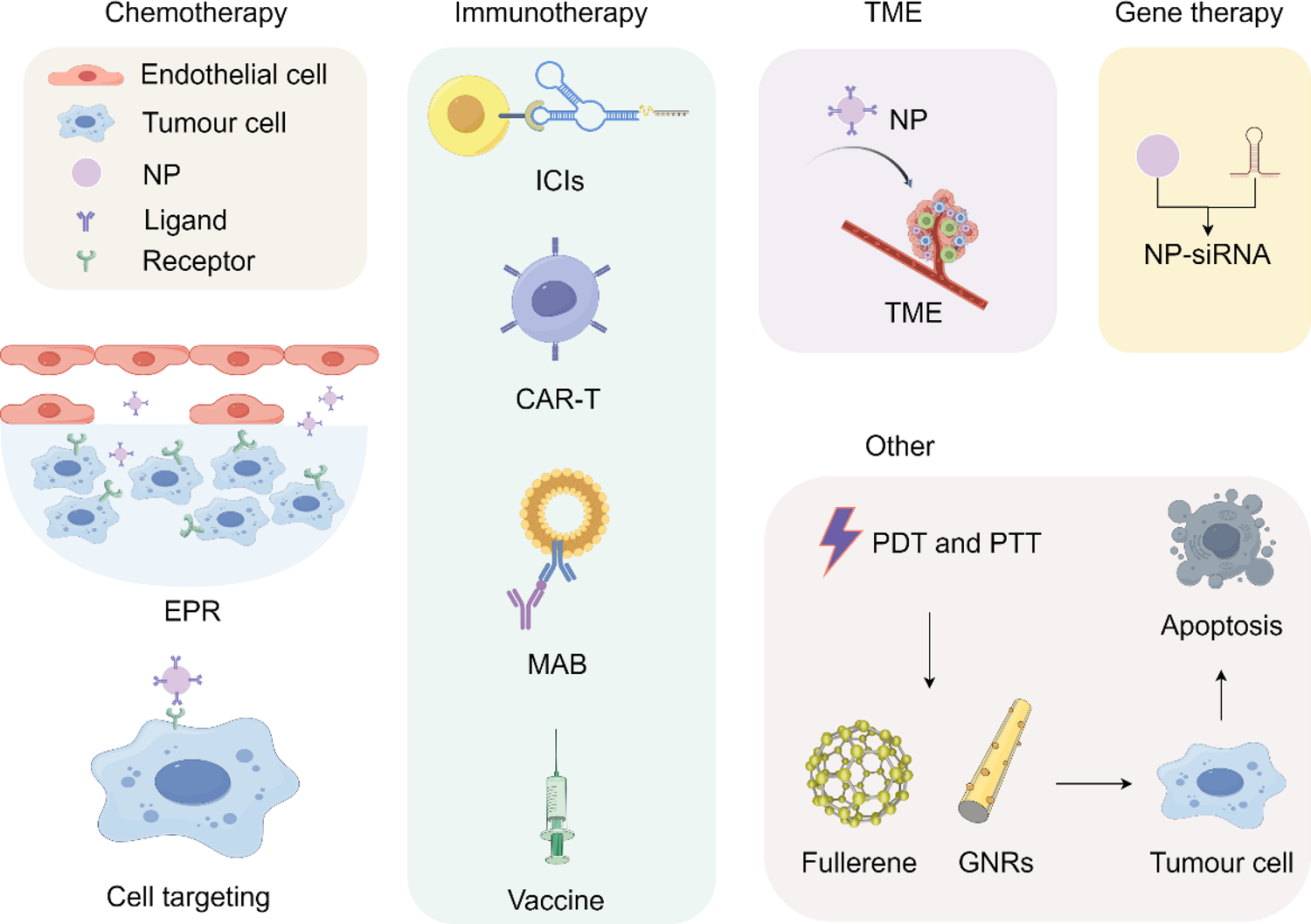
Nano-formulations and cancer treatment methods

Compared to normal tissues, tumor tissues are distinguished by an abundance of newly formed, irregular blood vessels, wider interstitial spaces, and inadequate lymphatic drainage. These characteristics enhance the EPR effect, thereby facilitating high permeability and retention of macromolecules and lipid particles [100]. In addition, tumor cells and vascular endothelial cell surfaces frequently overexpress specific receptors, such as programmed cell death ligand 1 (PD-L1) and CD47. By conjugating specific ligands of these receptors, such as monoclonal antibodies, antibody fragments, peptides, and growth factors, to the surface of nano-formulations, active targeting of nano-formulations can be achieved [101]. Notably, this active targeting strategy leverages the EPR effect as its foundation. Therefore, anticancer drugs can be delivered to tumors via both passive tissue targeting and active cell-targeted approaches. Currently, nano-formulations have ushered in an unprecedented new era in cancer therapy through various approaches including chemotherapy, immunotherapy, modulation of the tumor microenvironment, gene therapy, and others (Fig. 7).

#### The role of nano-formulations in chemotherapy

**Fig. 8:**
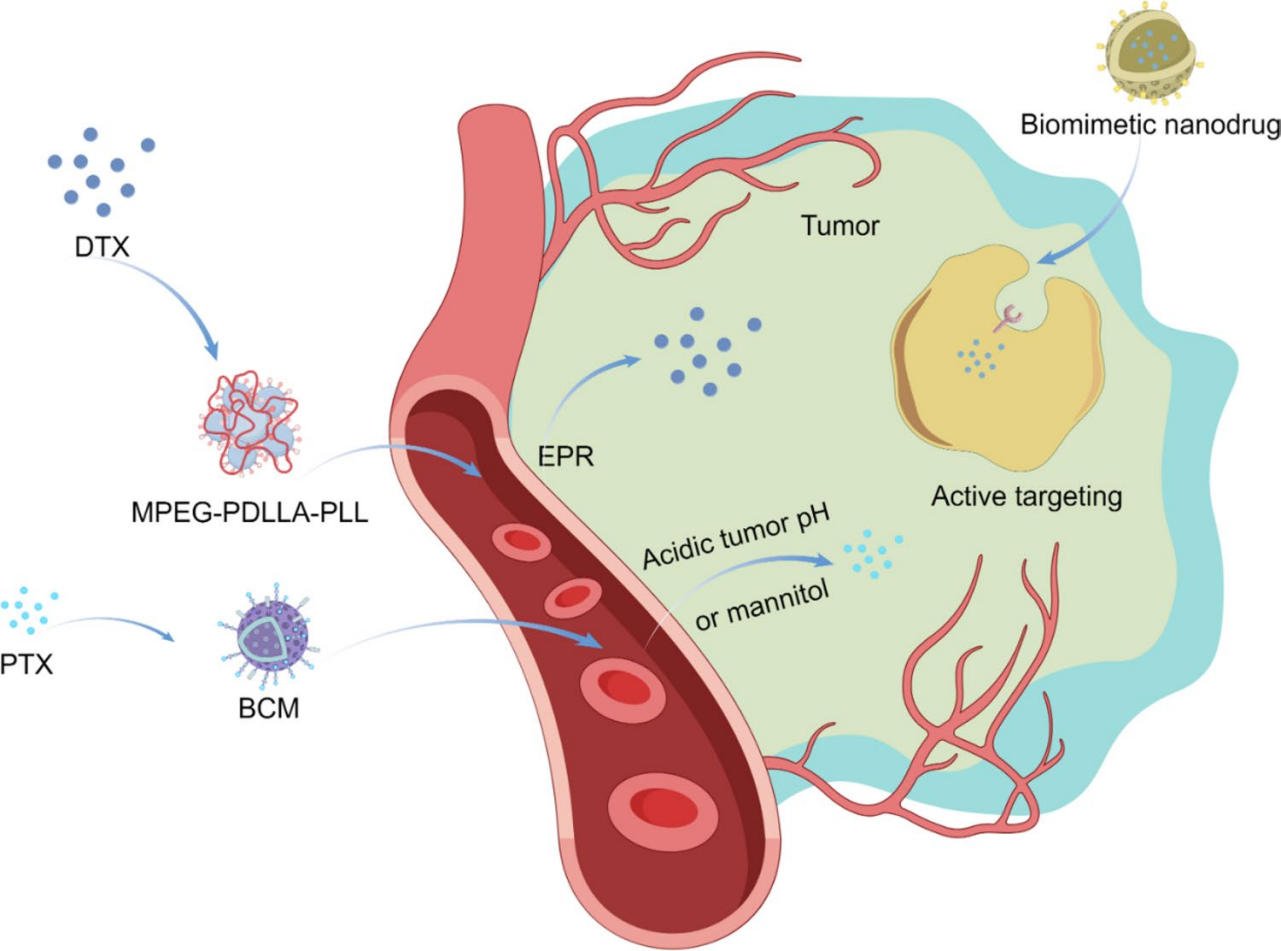
Nano-formulations in chemotherapy

In the realm of cancer chemotherapy, nano-formulations are emerging as game-changers, offering unprecedented advantages and innovations (Fig. 8). The EPR effect facilitates the passive accumulation of nano-formulation in tumor tissues, thereby mitigating the side effects associated with chemotherapeutic agents. Targeted nano-formulations can selectively accumulate in tumor tissues, thereby enhancing therapeutic efficacy while minimizing the toxic and side effects associated with chemotherapy drugs. Taxanes, as prototypical microtubule-stabilizing agents, are widely employed in the treatment of breast cancer, lung cancer, and various other malignancies [102]. Docetaxel (DTX), a member of this family, frequently encounters limitations in clinical application owing to its poor solubility, non-specific distribution, and rapid clearance.

Tan et al*.* have developed a series of novel triblock copolymers, MPEG-PDLLA-PLL, by introducing hydrophilic poly (L-lysine) (PLL) segments of different molecular weights into the monomethoxy poly (ethylene glycol)-poly (D, L-lactide) (MPEG-PDLLA) block copolymer [103]. The MPEG2k-PDLLA4k-PLL1k molecular chain could encapsulate up to 20% (w/w) DTX. The PDLLA segment (with a molecular weight of 4 kDa) forms a densely packed hydrophobic core, effectively encapsulating hydrophobic DTX molecules via hydrophobic interactions. This reduces drug release during blood circulation and prolongs the in vivo circulation time. The hydrophilic MPEG outer layer minimizes plasma protein adsorption and MPS clearance, thereby extending the micelles' residence time in the bloodstream. The positively charged PLL segment facilitates endocytosis by electrostatically interacting with negatively charged phospholipids in the tumor cell membrane, thus enhancing the intracellular delivery efficiency of DTX. The drug-loaded micelles exhibited high stability and demonstrated significantly enhanced inhibition of tumor cell growth, exhibiting superior anti-breast cancer efficacy. Xiao et al*.* have developed a novel borate cross-linked micelle (BCM) for loading paclitaxel (PTX) [104]. BCM demonstrated remarkable stability in both serum and plasma, exhibiting a significantly longer in vivo circulation time compared to non-cross-linked micelles (NCM). It released drugs in acidic environments or under the action of mannitol, and accumulated in tumors. In a mouse model of ovarian cancer, BCM-PTX preferentially accumulated in the tumor sites with a prolonged retention time, demonstrating superior therapeutic efficacy compared to NCM and PTX alone, while mice could tolerate higher doses of PTX.

It is important to highlight that in the majority of patients with advanced cancer, the EPR effect has not shown the anticipated therapeutic outcomes [105, 106]. Given this context, the development of nano-formulations with tumor-targeting capabilities to optimize the delivery of anticancer drugs is of particular importance. Wang et al*.* designed polymer micelles composed of 2-hydroxypropyl methacrylamide (HPMAm) and functionalized with biotin for the targeted delivery of PTX. Biotin receptor-mediated endocytosis facilitated the NPs to exhibit enhanced cytotoxicity of PTX in A549 lung cancer cells [107]. Heat shock protein 90 (HSP90), which is markedly overexpressed in a variety of cancers, facilitates the maturation of multiple oncogenic proteins and thereby promotes cancer cell proliferation [108]. Jia et al*.* designed HSP90 and CD44-targeted A6 peptide-functionalized biomimetic NPs (A6-NP) for the delivery of the HSP90 inhibitor G2111 [109]. A6-NP is straightforward to prepare, demonstrates excellent biocompatibility, and facilitates the controlled release of G2111, thereby minimizing off-target toxicity. A6-NP exhibits remarkable targeting capability and anticancer efficacy against hematological malignancies and colon cancer both in vitro and in vivo.

Furthermore, biomimetic membrane-based nano-formulations have been extensively utilized for the delivery of chemotherapy drugs. Cellular membranes as nano-formulations have attracted considerable attention for their capability to evade clearance by the reticuloendothelial system (RES) and facilitate precise tumor-targeted delivery [110]. For instance, Li et al*.* developed a biomimetic nanodrug delivery system for the targeted therapy of colorectal cancer, comprising a PTX-loaded gelatin nanogel core encapsulated within an HT-29 tumor cell membrane shell [111]. This system featured a core–shell nanostructure, which effectively minimized premature drug release and achieved selective targeting. Consequently, it significantly enhances the accumulation of PTX at the tumor site, markedly inhibits tumor growth, and demonstrates minimal side effects.

#### The role of nano-formulations in immunotherapy

Cancer immunotherapy leverages the body's immune system to elicit a robust anti-tumor response, thereby mitigating tumor evasion. It has emerged as an indispensable and transformative approach in cancer treatment [112]. Immunotherapy encompasses immunosuppressive therapy, adoptive cell therapy (ACT), tumor vaccine therapy, oncolytic virotherapy, and monoclonal antibody (MAB) therapy [113]. These therapies exhibit reduced side effects compared to conventional radiotherapy and chemotherapy, thereby enabling extended progression-free survival (PFS) and overall survival (OS) for patients. However, every coin has two sides. An overly active immune system can lead to severe adverse reactions, including immune-mediated inflammatory responses, autoimmune diseases, opportunistic infections, demyelinating disorders, and hypersensitivity reactions [114, 115]. This undoubtedly poses additional challenges on the path to treatment. In this context, nano-formulations shine like a beacon, illuminating a new direction for cancer immunotherapy.

**Fig. 9:**
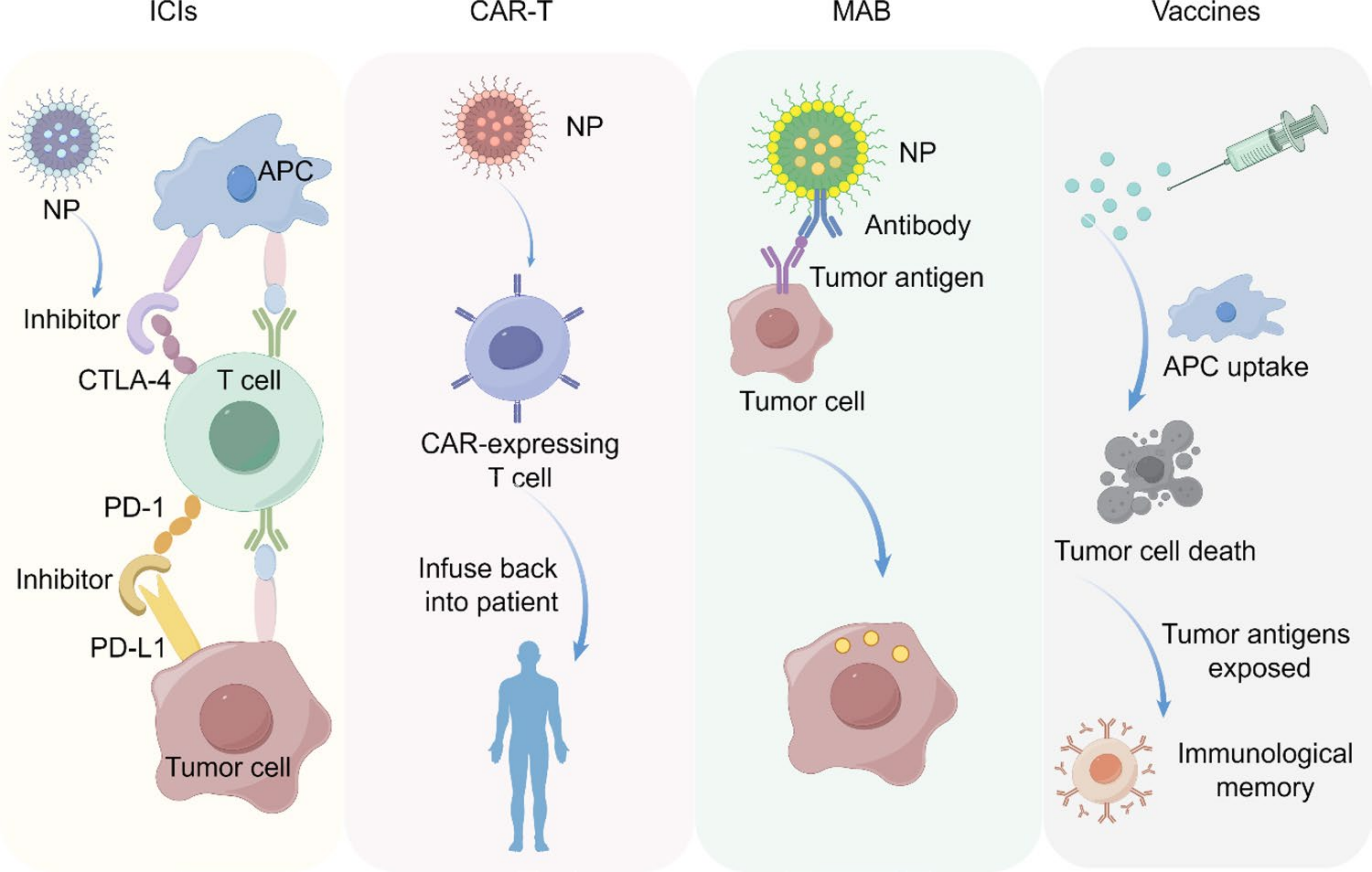
Nano-formulations in immunotherapy

Nano-formulations can enhance the efficacy of tumor immunotherapy, offering a potentially safer and more effective therapeutic approach [116, 117]. Many immunotherapeutic drugs struggle to achieve optimal efficacy due to their hydrophobicity, but nano-formulations can substantially improve the solubility and bioavailability of these drugs, thereby enhancing their therapeutic efficacy. Even more excitingly, nano-formulations exhibit sustained-release and controlled-release properties, which are crucial for achieving continuous dosing and prolonged therapeutic responses in tumor immunotherapy. Furthermore, nano-formulations can intelligently respond to the unique physiological characteristics of the tumor microenvironment. For instance, smart responsive nano-formulations, such as temperature-sensitive and pH-sensitive carriers, can flexibly adjust drug release according to changes in the tumor microenvironment. This "tailor-made" treatment approach demonstrates tremendous application potential. Currently, the application of nano-formulations in tumor immunotherapy is in full swing (Fig. 9).

##### Immune checkpoint inhibitors (ICIs)

T cells serve as the primary effector cells in tumor elimination, while ICIs can inhibit T cell proliferation and compromise their functionality [118]. Programmed cell death protein 1 (PD-1) and its ligand, PD-L1, serve as critical immune checkpoint molecules [119]. PD-1 inhibitors, such as pembrolizumab, have achieved significant efficacy in treating a range of malignancies, including NSCLC, melanoma, and hodgkin lymphoma (HL) [120]. These drugs function by inhibiting the interaction between PD-1 and PD-L1, thereby releasing T cells from the suppressive effects exerted by tumor cells and reinstating their antitumor activity. Cytotoxic T-lymphocyte-associated protein 4 (CTLA-4) is also a crucial immune checkpoint that functions to downregulate immune responses [121]. CTLA-4 inhibitors, such as ipilimumab, enhance antitumor immune responses by blocking the inhibitory signaling pathways of immune cells [122].

Dual ICI therapy, which combines two immune checkpoint inhibitors, has achieved significant results in various types of tumors. For instance, the combination therapy of Nivolumab (a PD-1 inhibitor) and Ipilimumab (a CTLA-4 inhibitor) has demonstrated remarkable efficacy in multiple tumor types, including melanoma, colorectal cancer (CRC), and NSCLC [123–125].

Protein hydrolysis-targeting chimeras (PROTACs) represent an innovative strategy for the selective degradation of proteins associated with tumors [126]. PROTACs generally comprise three key components: a small molecule that specifically binds to the protein of interest (POI), an E3 ligase ligand, and a linker [127]. However, challenges such as insufficient cellular internalization hinder PROTACs from effectively reaching their target sites and exerting their intended effects [128]. Based on these findings, researchers have engineered pH/cathepsin B sequentially responsive NPs (PSRNs) conjugated with PROTACs targeting cyclin-dependent kinases 4 and 6 (CDK4/6), leveraging ultra-pH-sensitive and enzyme-responsive nanotechnology [129]. PSRNs retain their nanostructure (approximately 40 nm) during circulation and accumulate in tumors via the EPR effect. In the acidic tumor microenvironment, they degrade into monomers (less than 10 nm), which enhances their tumor penetration and cellular internalization capabilities. Subsequently, PROTAC is released through enzymatic action. PSRNs not only enhance the degradation of target proteins both in vitro and in vivo in colorectal cancer (CRC) but also augment the efficacy of immune checkpoint blockades (ICBs) by upregulating PD-L1 expression in cancer cells and inhibiting regulatory T cell proliferation within the tumor microenvironment, thereby achieving enhanced anti-tumor effects in the CT26 tumor model [129].

##### CAR-T

ACT is an advanced immunotherapy approach that entails the isolation of autologous or allogeneic immune cells, followed by their activation or genetic modification in vitro. This process aims to enhance the antitumor efficacy of these cells before reinfusion into patients. This process is designed to expand immune cells that exhibit antitumor activity, which are subsequently reinfused into the patient to augment their antitumor immune response. ACT includes a variety of approaches, with chimeric antigen receptor T cell immunotherapy (CAR-T) receiving considerable attention in recent years.

CAR-T cell therapy is an innovative approach that involves genetically engineering patients' own T cells to express chimeric antigen receptors (CARs), which are designed to specifically recognize tumor-associated antigens [130]. These engineered T cells are subsequently expanded ex vivo and reinfused into the patients. The CAR-expressing T cells can specifically recognize and bind to target cell surface molecules, leading to the destruction of these cells. CAR-T cell therapy has demonstrated significant efficacy in the treatment of hematological malignancies and select solid tumors [130, 131].

Researchers have conjugated protein nanogels (NGs) loaded with the interleukin-15 superagonist complex (IL-15Sa) to the surface of T-cells, while simultaneously facilitating the fusion of these carrier cells into solid tumors [132]. Subsequent activation of the T-cell receptor (TCR) triggered the release of IL-15SA adjuvants, leading to a 16-fold increase in T-cell expansion relative to systemically injected IL-15SA.This significantly enhances the tumor-suppressing effect. The anti-Tcrl-15SA nanogel backpack method has significantly improved the efficacy and safety of human CAR-T cell therapy in treating mouse tumors [132]. Recently, researchers have engineered a biohybrid consisting of IL-12 nanostimulant-integrated CAR T cells (INS-CAR T). In the presence of tumor antigens, the increased thiol groups on the cell surface induce the release of IL-12 from the INS-CAR T biohybrid. This, in turn, promotes the secretion of chemokines (CCL5, CCL2, and CXCL10), which selectively recruit and expand CD8^+^ CAR T cells within the tumor. The IL-12 nanochaperone significantly enhances the antitumor capabilities of CAR T cells, leading to the elimination of solid tumors with minimal side effects [133].

##### MAB

MAB therapy inhibits tumor growth by directly binding to tumor antigens or modulating cell signaling pathways. In targeted cancer therapy, monoclonal antibodies are extensively utilized owing to their high specificity and potent anti-tumor efficacy [134]. Epidermal growth factor receptor (EGFR), prostate-specific membrane antigen (PSMA), and human epidermal growth factor receptor 2 (HER2) have emerged as three prominent targets for MAB-functionalized NPs in cancer therapy, which have been extensively investigated in recent years [134–136]. By conjugating NPs loaded with chemotherapy or radiotherapy agents to monoclonal antibodies, tumor cells can be specifically targeted and attacked [134], thereby achieving enhanced specificity and reduced toxicity. Researchers have prepared rapamycin-loaded poly (lactic-co-glycolic acid) (PLGA) NPs (NPGA) with EGFR antibodies conjugated to their surfaces. This modification resulted in a more than 13-fold increase in uptake by MCF-7 cells compared to unconjugated NPs, thereby significantly enhancing the antitumor efficacy of the drug [137]. Saniee et al*.* conjugated glutamic acid-urea-lysine, a PSMA enzyme inhibitor, with poly (lactic-co-glycolic acid)-polyethylene glycol (PLGA-PEG) NPs for the targeted delivery of docetaxel to prostate cancer cells, thereby enhancing the antitumor efficacy of docetaxel [138]. To further enhance the therapeutic efficacy of anticancer drugs, MAB can be conjugated with cytotoxic agents, forming antibody–drug conjugates (ADCs) [139]. Trastuzumab (Herceptin^®^) is a MAB used for the treatment of breast cancer with HER2 positive expression. Studies have been conducted using trastuzumab (Tmab) in ADC systems, and the results have shown improved therapeutic effects compared to the use of Tmab alone [140]. Researchers have conjugated Tmab to PLGA-PEG/PLGA NPs encapsulating docetaxel, facilitating sustained, controlled, and targeted release of docetaxel specifically into breast cancer cells [141]. Recently, researchers have developed a nano-formulation comprising a PTX-loaded core and a surface modified with Tmab. Experimental results demonstrate that the novel NP complex exhibits superior antitumor efficacy compared to PTX or Tmab alone, with relatively lower cytotoxicity observed in human breast epithelial cells used as a control group for the NP complex [142].

##### Nano-vaccines

Nano-vaccines leverage nanomaterials as carriers for the targeted delivery of specific antigens and adjuvants, demonstrating significant efficacy in disease prevention and treatment, particularly in influenza prophylaxis and cancer therapy. Tumor vaccines constitute an immunotherapeutic strategy that activates the patient's own immune system to eliminate or control tumor growth. The size of NPs, comparable to that of pathogens, facilitates efficient uptake by antigen-presenting cells (APCs).

Recently, researchers have developed a hybrid mRNA delivery platform (MnLNPs) by co-assembling manganese oxide NPs (Mn_3_O_4_ NPs) with lipid NPs (LNPs). This innovative approach aims to enhance the efficacy of mRNA vaccines [143]. Mn_3_O_4_ NPs exhibited potent capabilities in scavenging ROS and generating oxygen, thereby increasing intracellular ATP levels and improving mRNA translation efficiency. Furthermore, Mn^2+^ activated the cGAS (cyclic GMP-AMP synthase)-STING (stimulator of interferon genes) pathway, thereby promoting dendritic cell (DC) maturation and type I interferon secretion. This activation subsequently enhanced both innate and adaptive immune responses. The combination of MnLNPs@mRNAE7 vaccine with anti-PD-L1 antibody (αPD-L1) has reprogrammed the immunosuppressive tumor microenvironment, further augmenting the antitumor effect [143].

Cytotoxic T lymphocytes (CTLs) initiate an immune response against tumor cells by recognizing tumor-associated antigen peptides presented by MHC-I molecules on the cell surface [143]. However, tumor antigens must be processed by antigen-presenting cells (such as dendritic cells) prior to presentation by MHC-I molecules. This process encompasses endocytosis, proteasomal degradation, and the loading of peptide fragments onto MHC-I molecules [144, 145]. The efficiency of antigen processing and presentation directly impacts the extent of CTL activation, consequently influencing the magnitude of the immune response. Tumor cells can evade immune system surveillance through multiple mechanisms, such as the downregulation of MHC-I molecule expression or the secretion of immunosuppressive factors [146]. Tumor vaccines are designed to augment the immune system's capability to recognize and target tumors through the presentation of tumor-specific antigens, thereby eliciting a more robust and specific antitumor immune response. Vaccines can activate the immune system, particularly CTLs, through various strategies such as the use of tumor cells, tumor antigen peptides, DNA, or mRNA [147]. Lipid NPs (LNPs) function as efficient vaccine delivery systems by enhancing antigen stability and promoting more effective uptake by immune cells [148].

Recently, researchers have developed an innovative tumor vaccine strategy termed TAgD-TVac. This approach harnesses targeted antigen degradation to augment antigen processing and cross-presentation. By pre-conjugating tumor antigens with E3 ubiquitin ligase ligands and encapsulating them within lymph node-targeting LNPs, TAgD-TVac successfully enhanced the degradation efficiency and cross-presentation of tumor antigens. This led to improved CTL activation and significantly inhibited tumor growth, metastasis, and recurrence in various cancer models, including melanoma, triple-negative breast cancer, Lewis lung cancer, and ovarian cancer. When used in conjunction with immune checkpoint inhibitors, a synergistically enhanced therapeutic effect was observed [149].

#### The role of nano-formulations in remoulding the tumor microenvironment (TME)

TME represents a complex ecosystem composed of tumor cells, immune cells, neuronal cells, vascular cells, and the extracellular matrix, playing a crucial role in tumor growth, invasion, metastasis, and response to treatment [150, 151]. Studies have demonstrated that the interaction between the nervous system and tumors significantly influences tumor development and progression. Specifically, the density of tumor-associated nerves is closely correlated with both tumor size and patient survival rates [152–154]. Tropomyosin receptor kinase (TRK), a receptor tyrosine kinase expressed in both the peripheral and central nervous systems, plays an essential role in promoting neuronal growth and regulating synaptic plasticity [155]. Activation of TRK in the TME not only promotes neuronal growth but also enhances the proliferation and migration of tumor cells. Tumor-associated macrophages (TAMs), which exhibit dual roles within TME, can polarize into either M1 (anti-tumor) or M2 (tumor-promoting) phenotypes. Notably, M2-polarized TAMs are closely associated with nerve infiltration and play a significant role in promoting tumor growth and facilitating immune evasion.

Although chemotherapy drugs such as gemcitabine are the primary means of treating pancreatic cancer, they may inadvertently promote neuronal growth, which can potentially support tumor progression. Outer membrane vesicles (OMVs), a type of nanocarrier with high tumor-targeting efficacy, not only activate immune responses but also modulate the polarization state of AMs [156]. Utilizing nano-formulations for the delivery of the TRK inhibitor larotrectinib can enhance its concentration within the TME while minimizing adverse effects on normal tissues.

Researchers have reported a novel nanocarrier, Lar@NP-OMVs, which integrates OMVs with the neuropeptide NP41 and encapsulates larotrectinib. Lar@NP-OMVs can efficiently target tumor-associated nerves, reducing neurite outgrowth by blocking the neurotrophic factor/Trk signaling pathway. Furthermore, through OMV-mediated polarization of TAMs, Lar@NP-OMVs not only induce neurotoxicity but also effectively inhibit the proliferation, migration, and angiogenesis of pancreatic cancer cells. Furthermore, the combination of Lar@NP-OMVs with gemcitabine mitigates the latter's promotion of nerve infiltration and neurite outgrowth, thereby enhancing the efficacy of chemotherapy [157].

#### The role of nano-formulations in gene therapy

**Fig. 10:**
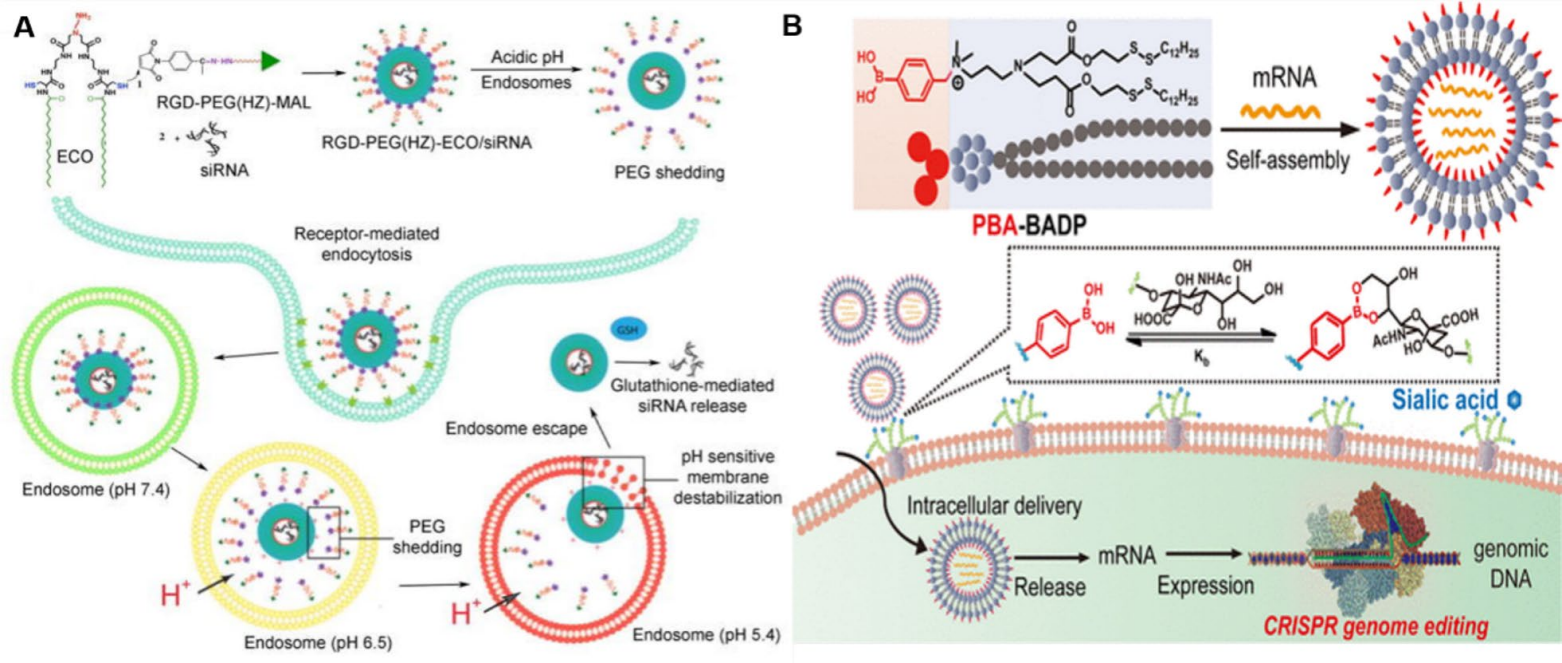
Schematic model of nano-formulations in gene therapy. **A** Design and schematic representation of pH-sensitive PEG(HZ)-ECO/siRNA dual nanoparticles. **B** Assembly schematic of PBA–BADP/mRNA nanoparticles for mRNA delivery and genome editing *Adapted from Gujrati M, Vaidya AM, Mack M, *et al*. Targeted Dual pH-Sensitive Lipid ECO/siRNA Self-Assembly Nanoparticles Facilitate *In Vivo* Cytosolic sieIF4E Delivery and Overcome Paclitaxel Resistance in Breast Cancer Therapy. Adv Healthc Mater. 2016;5(22):2882–95. Tang Q, Liu J, Jiang Y, et al. Cell-Selective Messenger RNA Delivery and CRISPR/Cas9 Genome Editing by Modulating the Interface of Phenylboronic Acid-Derived Lipid Nanoparticles and Cellular Surface Sialic Acid. ACS Appl Mater Interfaces. 2019;11(50):46585-90*

Nano-formulations serve as crucial vehicles in gene delivery and are essential non-viral vectors for gene therapy applications, as presented in Fig. 10. By using nanotechnology, therapeutic genes or gene vectors can be accurately delivered to target cells or tissues for gene correction, gene amplification, gene inactivation or immunotherapy [158]. This approach is characterized by accurate targeting, high-efficiency delivery, minimal toxicity, and superior biocompatibility. siRNA can induce sequence-specific cleavage of mRNA molecules, resulting in their degradation. This mechanism is widely employed for the silencing or downregulation of gene expression. siRNA can be delivered through various nano-formulations, including LNPs, metal NPs, and silicon dioxide NPs, among others [158]. eIF4E, functioning as an oncogene, is implicated in tumorigenesis upon its overexpression or activation. Researchers have designed and developed a dual pH-sensitive LNP that can effectively inhibit eIF4E, thereby restoring the sensitivity of triple-negative breast tumors to paclitaxel treatment [159]. Messenger RNA (mRNA) constitutes a category of nucleic acid-based therapies employed for genome editing and the treatment of genetic disorders. Among these tools, CRISPR/Cas9 emerges as an exceptionally powerful and precise instrument for genome editing. By precisely modulating the interaction between phenylboronic acid (PBA)-derived LNPs and sialic acid (SA) on the cell surface, researchers have successfully delivered CRISPR/Cas9 into cancer cells, enabling selective genome editing. Experimental findings demonstrate that PBA-BDAP possesses the ability to specifically target and deliver P53 mRNA to cancer cells, thereby exerting an inhibitory effect on tumor cell proliferation [160].

Exosomes are lipid-encapsulated vesicles that are rich in lipids, nucleic acids, proteins, and other bioactive molecules. As natural nano-formulations for therapeutic drug delivery, exosomes have been extensively studied. They can deliver a wide range of therapeutic agents, including small-molecule anticancer and anti-inflammatory drugs, proteins, and nucleic acids such as siRNA and miRNA [161]. Owing to the presence of lipids and molecules on the exosome membrane that resemble those of their parent cells, exosome NPs are capable of evading immune surveillance and efficiently internalizing into target cells [162].

Researchers utilized exosomes loaded with doxorubicin (DOX) (exoDOX) to treat human breast cancer cells. Experimental results demonstrated that, compared to free DOX, exoDOX not only enhanced the cytotoxicity of doxorubicin but also but also effectively reduced drug accumulation in the heart [163]. Recently, researchers have demonstrated the significant antitumor efficacy of cholesterol-rich exosomes in delivering PLK1 siRNA in colorectal cancer models. The cholesterol-enriched milk-derived exosomes (MEs), with a 30% cholesterol enhancement, directly release siRNA via membrane fusion, thereby significantly inhibiting tumor growth, promoting cell apoptosis, and reducing tumor metastasis. Moreover, experimental evidence has shown that MEs exhibit favorable in vivo safety profiles and low immunogenicity, indicating their potential value in cancer therapy [164].

#### The role of nano-formulations in other therapies

Photodynamic therapy (PDT) and PTT have emerged as promising cancer treatment modalities in recent years, both of which are categorized under phototherapy [162]. PDT entails the targeted accumulation of photosensitizers in tumor regions via specialized methods. Subsequently, upon irradiation with light of a specific wavelength, the photosensitizers become activated, producing singlet oxygen and other cytotoxic ROS [165]. These reactive species effectively induce apoptosis or necrosis in tumor cells, thereby achieving the therapeutic objective. In comparison, PTT emphasizes the use of materials with high photothermal conversion efficiency. When exposed to light, these materials efficiently convert light energy into thermal energy, leading to a substantial increase in the temperature of the targeted tumor region [166]. The high-temperature environment is lethal to cancer cells, inducing their apoptosis and thereby enhancing therapeutic efficacy.

To enhance anti-tumor efficacy, a synergistic approach combining siRNA drugs with PTT is employed for the treatment of prostate cancer cells. In particular, gold nanorods (Au NRs) functionalized with zinc(II)-dipicolylamine are utilized as carriers for siPLK, which specifically inhibits the expression of polo-like kinase-1 (PLK1) in cancer cells, thereby forming innovative NPs. These NPs effectively inhibit PLK1, thereby reducing the viability and proliferative activity of cancer cells. Moreover, the NPs demonstrate pronounced anti-tumor efficacy upon laser irradiation in both PC-3 cell lines and PC-3 tumor-bearing mice models [167]. Fullerenes, as a type of nanomaterial, exhibit outstanding performance in both PDT and PTT [168, 169]. Researchers have developed a type of near-infrared light-excited NP that leverages photoacoustic imaging to achieve both photothermal and PDT effects on tumors. Compared to fullerenes and DOX, this NP demonstrates superior ROS generation and heat production. Both in vitro and in vivo studies have consistently shown that the synergistic combination of PDT and PTT effectively inhibits tumor growth [169].

Currently, it is gratifying to note that clinical trials of nano-formulations for tumor treatment are currently underway (Table 1).

**Table 1:** Clinical trials for cancer treatment via nano-formulations

Name	Description	Nanocarrier	Indication	Status	Ref
CYL-02	CYL-02 plus gemcitabine	Polyethyleneimine	Pancreatic ductal adenocarcinoma	Phase 2	NCT02806687
Nano-SMART	AGuIX Gadolinium-based NPs with stereotactic magnetic eesonance-guided adaptive radiation therapy	AGuIX	Pancreatic and Lung Cancer	Phase 1/2	NCT04789486
Pembrolizumab(Pbr)/Nab-Paclitaxel	Pbr/Nab-Paclitaxel followed by Pbr/Epirubicin/Cyclophosphamide	Nab-paclitaxel	Triple negative breast cancer	Phase 2	NCT03289819
BCMA nano-antibody CAR-T cells	Study the safety and efficacy of BCMA nano-antibody CAR-T in MM therapy	Nano-antibody	Multiple myeloma	Phase 1	NCT03661554
Nano-Quercetin	Quercetin versus its encapsulated NPs	PLGA-PEG NPs	Squamous cell carcinoma	Phase 2	NCT05456022
Oral Nanocurcumin	Adjuvant therapy	NPs	Anogenital warts	Phase 2/3	NCT06281353

### Nano-formulations and drug resistance

**Fig. 11:**
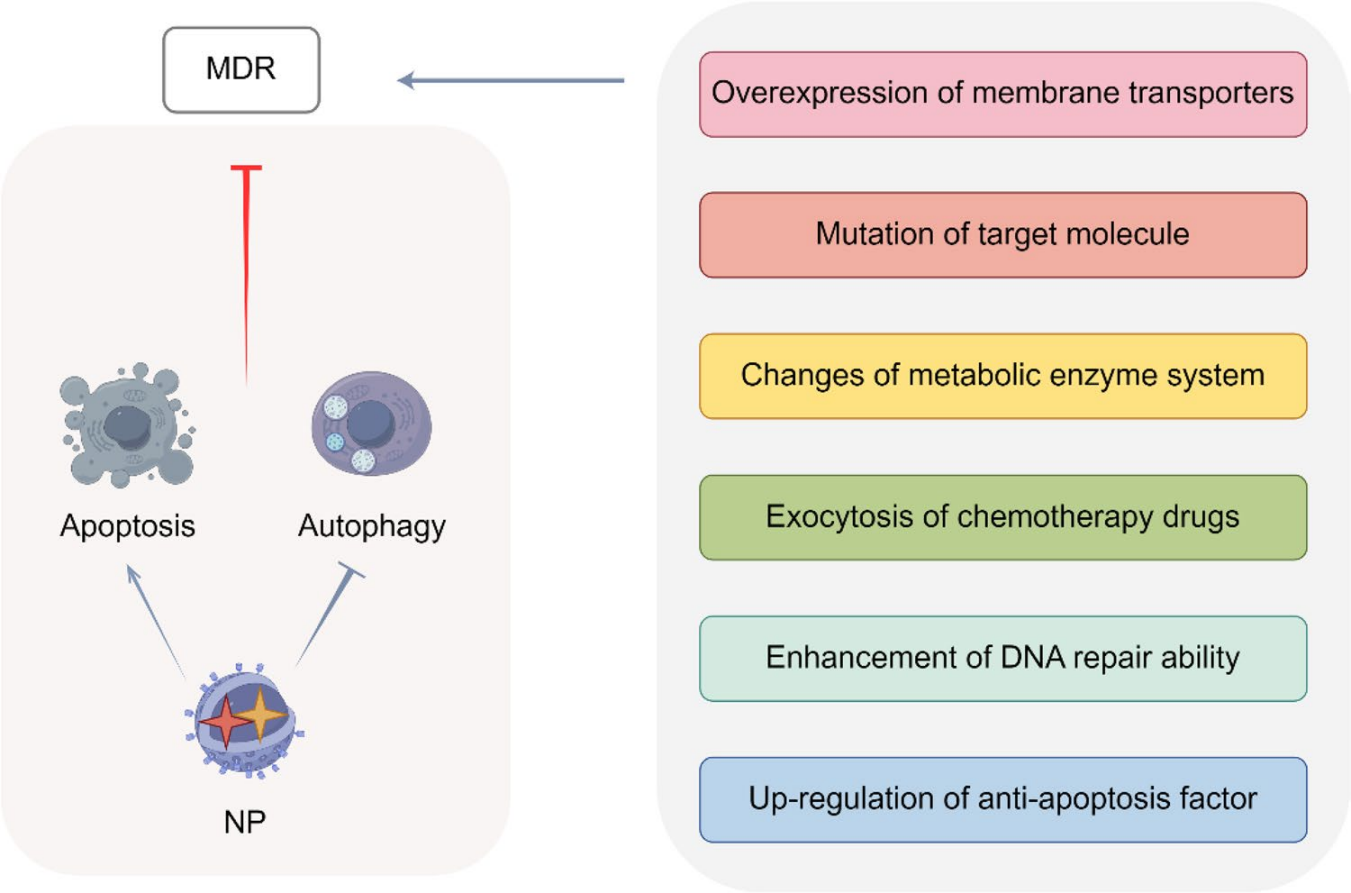
Mechanisms of nano-formulations and drug resistance

**Fig. 12:**
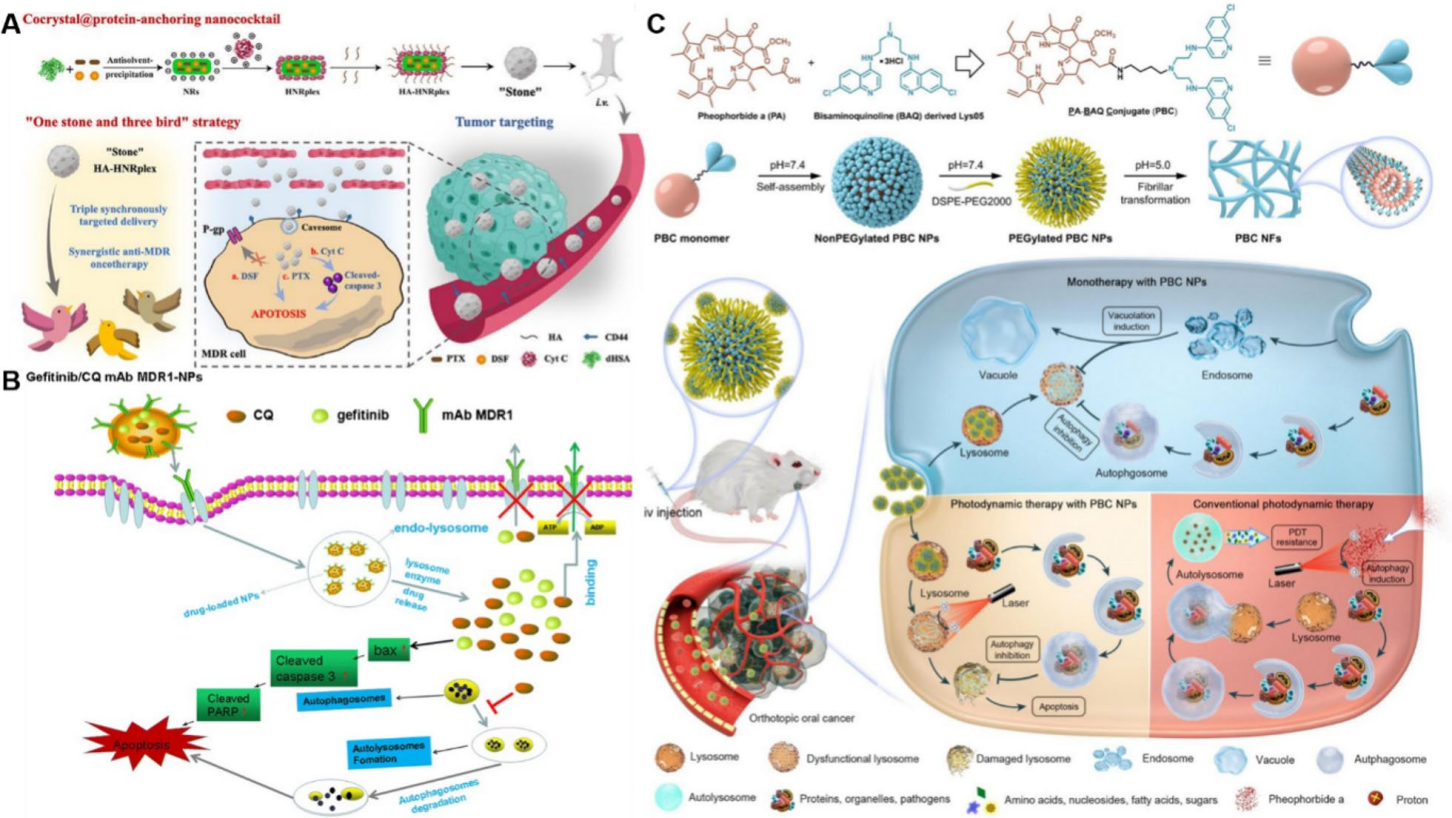
Schematic model of nano-formulations and multidrug resistance. **A** The triple-payload delivery platform (HA-HNRplex) adopts a "kill three birds with one stone" strategy, enabling synchronous delivery of PTX, DSF, and Cyt C for the treatment of multidrug-resistant cancers. **B** Monoclonal antibody MDR1-modified chitosan nanoparticles (CNPs) can overcome acquired resistance to EGFR-tyrosine kinase inhibitors (EGFR-TKIs) across various antitumor targets. **C** Chemical structure, transformation behavior, and schematic representation of a lysosomal pH-responsive small-molecule-based nanotransformer designed to overcome autophagy-induced drug resistance in cancer *Adapted from Zou J, Xing X, Teng C, *et al*. Cocrystal@protein-anchoring nanococktail for combinatorially treating multidrug-resistant cancer. Acta Pharm Sin B. 2024;14(10):4509–25. Zheng Y, Su C, Zhao L, *et al*. mAb MDR1-modified chitosan nanoparticles overcome acquired EGFR-TKI resistance through two potential therapeutic targets modulation of MDR1 and autophagy. J Nanobiotechnology. 2017;15(1):66. Ma Z, Lin K, Tang M, *et al*. A pH-Driven Small-Molecule Nanotransformer Hijacks Lysosomes and Overcomes Autophagy-Induced Resistance in Cancer. Angew Chem Int Ed Engl. 2022;61(35):e202204567*

Despite remarkable progress in the development and application of chemotherapy drugs, multidrug resistance (MDR) continues to pose a significant barrier to effective treatment. Repeated or multiple administrations of chemotherapeutic drugs can lead to the emergence of MDR in tumor cells, thereby diminishing therapeutic efficacy [170]. MDR refers to the phenomenon where tumor cells acquire resistance not only to a specific antitumor drug but also exhibit cross-resistance to multiple other antitumor agents with distinct structures and mechanisms of action [171]. The mechanisms underlying MDR primarily include six aspects (Fig. 11): (i) overexpression of membrane transporter proteins, specifically ATP-binding cassette (ABC) proteins, which reduces drug influx and increases drug efflux in tumor cells; (ii) mutations in the target molecules of drug action that prevent drug binding; (iii) alterations in the metabolic enzyme system that either inactivate drugs or convert them into non-toxic forms; (iv) sequestration of chemotherapeutic drugs within vesicles by resistant tumor cells, followed by their expulsion through exocytosis; (v) enhanced DNA repair capabilities in tumor cells that confer resistance to chemotherapeutic drugs; (vi) upregulation of anti-apoptotic factors in tumor cells to inhibit drug-induced apoptosis [172, 173]. By modulating multiple signaling pathways simultaneously, NP co-delivery therapy shows significant potential in overcoming MDR, thereby enhancing the efficacy of disease treatment, as presented in Fig. 12.

Zheng et al*.* prepared chitosan NPs conjugated with MDR1 antibodies, encapsulating both gefitinib and the autophagy inhibitor chloroquine [174]. These NPs specifically bind to cells overexpressing MDR1, thereby enhancing their internalization. By modulating autophagy and concurrently inhibiting MDR1-mediated drug efflux, the intracellular concentration of gefitinib is significantly increased. Recently, Zou and co-workers developed a nano-formulation (HA-HNRplex) with a triple payload of MDR inhibitor (DSF), biological macromolecule apoptosis promoter (Cyt C), and PTX through the cocrystal@protein anchoring strategy [172]. HA-HNRplex exhibited high encapsulation efficiency and induced the apoptosis in MDR cells through a cascade mechanism that includes inhibition of P-gp, an ABC transporter implicated in MDR, elevation of intracellular cytochrome C levels, and upregulation of cleaved caspase-3 expression. HA-HNRplex demonstrates anti-MDR and anti-tumor effects on both normal and drug-resistant tumor cells, effectively treating cancer in A549/Taxol drug-resistant tumor-bearing mice.

Autophagy is a critical lysosome-dependent pathway responsible for recycling damaged macromolecules and organelles, which ultimately contributes to cancer cell survival and treatment resistance [175]. With the deepening understanding of the mechanisms underlying multidrug resistance (MDR), researchers have initiated explorations into lysosome-targeted MDR treatment strategies. By modulating lysosomal function or inhibiting specific active substances within them, it is possible to influence the formation and progression of MDR mechanisms, thereby enhancing the efficacy of chemotherapy drugs and mitigating drug resistance. Ma et al*.* have reported a nonpeptide PBC (pheophorbide a-bisaminoquinoline conjugate) targeting lysosomes [176]. At physiological pH, PBC self-assembles into NPs and subsequently transforms into nanofibrils within the lysosomes of tumor cells. This transformation resulted in lysosomal dysfunction, inhibition of autophagy, and abnormal cytoplasmic vacuolation. Furthermore, PBC-mediated photodynamic therapy could overcome the therapeutic resistance induced by intrinsic autophagy that is inherent in traditional photodynamic therapy.

## Nano-formulations: putative strategies to treat pulmonary disease

Pulmonary disease, such as chronic obstructive pulmonary disease (COPD) and asthma, significantly impair patients' quality of life [177]. The treatment of respiratory diseases poses significant challenges, primarily due to insufficient dosing of drugs delivered to the respiratory tract or the inadequate targeting capability of conventional medications in reaching the affected areas [178, 179].

**Fig. 13:**
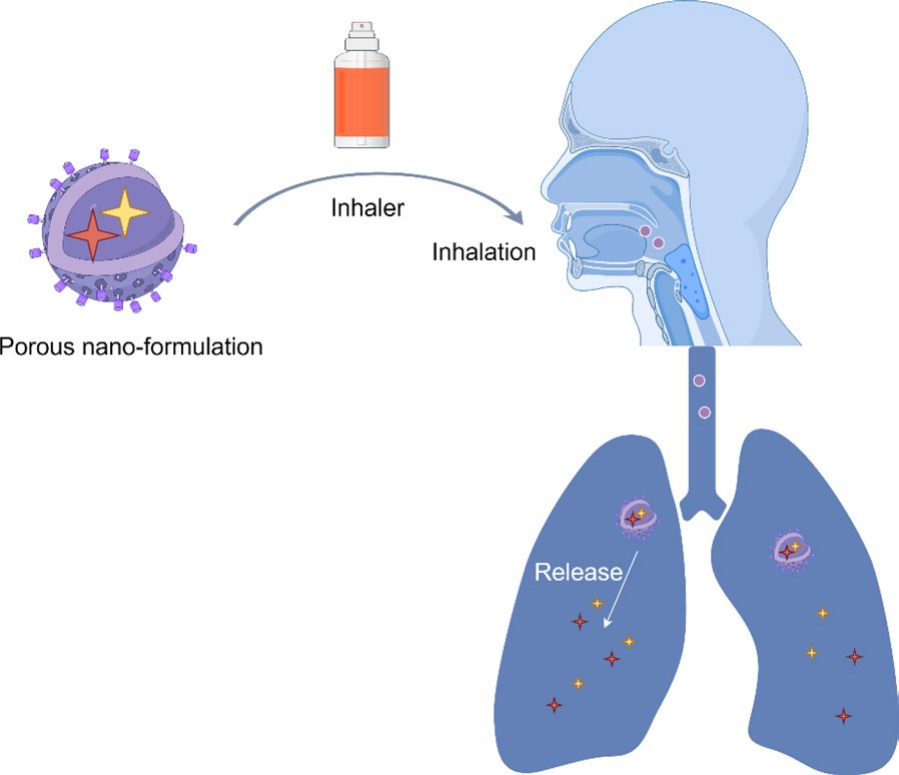
Nano-formulations for treating pulmonary diseases

Inhalable nano-formulations have exhibited remarkable advantages and considerable potential in the treatment of lung diseases (Fig. 13). They can overcome physiological barriers and enhance the deposition efficiency of aerosols in the lungs, thereby achieving precise drug delivery to the diseased sites within the lungs [178, 180]. Drug formulations developed via nanotechnology can enhance drug solubility, improve stability and bioavailability, thereby significantly boosting therapeutic efficacy [181]. Nano-formulations facilitate targeted drug delivery, thereby reducing the required dosage and minimizing both toxicity and side effects associated with the drugs [182, 183]. The sole inhalable nano-formulation currently available on the market is amikacin liposome inhalation suspension (Arikayce®), which received FDA approval in 2018 [184]. Arikayce® utilizes charge-neutral liposomes to encapsulate amikacin, thereby protecting the drug from rapid clearance by alveolar macrophages and extending its retention time in the lungs [185]. Via the eFlow nebulization system, Arikayce^®^ ensures effective deposition of the drug at the site of infection [185]. In the pulmonary environment, amikacin is released in a controlled and sustained manner, which helps to maintain therapeutic drug concentrations in the lungs, minimize systemic exposure, and consequently reduce the risk of systemic toxicity. A few inhalable nano-formulations are being developed for clinical trials (Table 2). Porous nano-formulations encapsulating docetaxel and celecoxib, fabricated via the emulsion-solvent evaporation method, exhibited significantly improved deposition efficiency in the lungs following particle size optimization. These findings indicate that this approach represents an effective and low-toxicity treatment option for lung tumors [186].

**Table 2:** Inhalable nano-formulations under clinical application

Drug	Nanocarrier	Indication	Status	Ref
Amikacin	Liposome	Cystic Fibrosis patients with chronic pseudomonas aeruginosa infection	Phase 3	NCT01316276
Amikacin	Liposome	Bronchiectasis	Phase 2	NCT00775138
Salbutamol Sulphate	Niosomes	Pulmonary disease	Phase 2	NCT03059017
Remdesivir (GS-5734) and NA-831 (NEUROSIVIR)	Inhaled NP	Severe acute respiratory syndrome	Phase 1	NCT04480333
PRS-060	Lipocalin-1	Mild asthma	Phase 1	NCT03574805

## Nano-formulations: promising treatment in the therapy for cardiovascular diseases

Cardiovascular diseases (CVD) are the foremost cause of mortality worldwide, presenting significant threats to human health and life [187]. CVD encompasses a range of conditions including, but not limited to, atherosclerosis, hypertension, and myocardial infarction (MI), all of which can result in tissue ischemia and potentially fatal outcomes. Nano-formulations have shown great potential in repairing various organ injuries [188]. Nano-formulations are exhibiting substantial advantages and considerable potential in the diagnosis and treatment of CVD.

### Application in molecular imaging

NPs exhibit a wide range of diagnostic applications in CVD, such as MRI, photoacoustic imaging, and computed tomography-positron emission tomography (CT-PET) imaging, among others [189]. As early as 1990, Weissleder et al*.* developed an ultra-small superparamagnetic iron oxide (USPIO) formulation for application in MRI [190]. Subsequently, biotin-modified liquid perfluorocarbon NPs, serving as a novel targeted ultrasound contrast agent, significantly enhanced the visualization of thrombosis [191, 192]. Furthermore, hyaluronic acid-polypyrrole NPs loaded with doxorubicin have been developed for pH-responsive activatable fluorescence imaging, whereas AuNRs are employed for CT imaging [193, 194].

Multimodal imaging signifies a significant advancement in the evolution of imaging technology. Nano-formulations are capable of efficiently integrating multiple imaging probes or contrast agents into a single system, thereby realizing a genuine multimodal imaging platform [195, 196]. Utilizing tobacco mosaic virus (TMV) as a carrier, Michael et al. developed a targeted multimodal nano-contrast agent by incorporating near-infrared dye Cy5, chelated gadolinium (Gd) ions, and VCAM-1 peptides. This agent enables simultaneous magnetic resonance and fluorescence imaging of atherosclerotic plaques [197]. Kwon et al*.* developed thrombin-activated fluorescent peptides (TAP) incorporated into silica-coated AuNPs (TAP-SiO₂@AuNPs). This innovation enables the clear differentiation between thrombosis and surrounding tissues via dual-mode NIRF/micro-CT imaging in a mouse model [198].

**Fig. 14:**
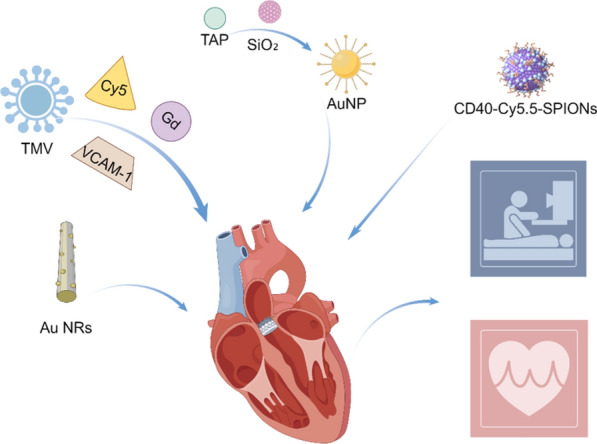
Nano-formulations for molecular imaging

Recently, Wu et al*.* developed a novel probe, CD40-Cy5.5-SPIONs, utilizing superparamagnetic iron oxide NPs (SPIONs) as the carrier to specifically target CD40 [199]. Upon intravenous administration into a mouse model of atherosclerosis, this probe demonstrated significant potential for MRI and optical dual-modality molecular imaging of vulnerable atherosclerotic plaques. In summary, the application of nano-formulations in molecular imaging has provided robust support for precise diagnosis and personalized medicine in cardiovascular diseases (Fig. 14).

### Atherosclerosis (AS)

**Fig. 15:**
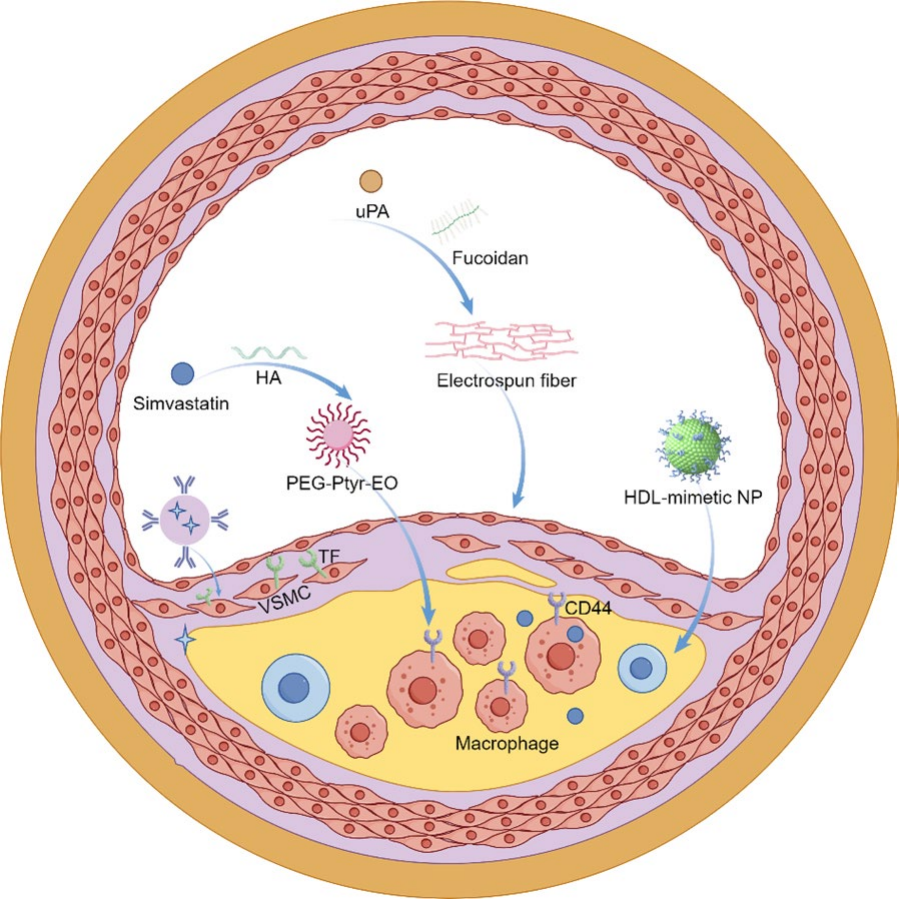
Nano-formulations for the therapy of AS

Atherosclerosis (AS) is characterized by the deposition of blood components, such as lipids, within the arterial intima, accompanied by the proliferation of smooth muscle cells and an increase in collagen fibers. This process leads to the formation of lipid-rich, necrotic lesions with a porridge-like appearance. As the disease progresses, the hardening of the blood vessel walls may occur, and inflammation may develop in response to plaque formation [200]. Nano-formulations, leveraging their unique targeting delivery and high-efficiency therapeutic properties, exhibit significant advantages in the treatment of AS (Fig. 15).

Various forms of nanodrug carriers, including liposomes, nano-micelles, and inorganic NPs, can specifically target and deliver drugs to atherosclerotic plaque sites, thereby effectively prolonging the plasma half-life of the drugs [201, 202]. The primary treatment methods encompass modulating lipoprotein levels, mitigating inflammation, reducing plaque area, and stabilizing vulnerable plaques [203, 204].

Wu et al*.* investigated a specific type of PLGA NPs conjugated with EGFP-EGF1, which can be efficiently internalized by vascular smooth muscle cells (VSMCs) overexpressing tissue factor (TF) in vitro and effectively targeted to atherosclerotic plaques in vivo [205]. The surface of ENP is covalently functionalized with the EGFP-EGF1 protein, which specifically binds to tissue factor (TF), thereby conferring targeting capability to atherosclerotic plaques on the nanoparticles. PLGA is used as the polymer material, which has good biocompatibility and degradability, ensuring the safety of the drug delivery system. The nano-formulation facilitates the gradual release of the drug at the plaque site, thereby maintaining therapeutic concentrations effectively, reducing systemic exposure, and minimizing potential side effects. Zhao et al*.* developed a design approach for peptide-lipid NPs that mimics the functionality of HDL, thereby facilitating efficient cellular cholesterol efflux and leading to a significant reduction in plasma total cholesterol levels in mice with atherosclerosis [206]. Multiple peptide chains significantly enhance peptide-lipid affinity, improving the stability of nanoparticles and their cholesterol-binding capacity. By mimicking the function of ApoA-I protein in natural HDL, they facilitate cholesterol efflux to small, lipid-poor HDL particles, which constitutes a critical step in reverse cholesterol transport. The surface charge and hydrophilicity of the particles are optimized through the incorporation of cholesterol and PEGylated lipids, thereby prolonging their circulation time in vivo and preventing clearance by the phagocytic system.

Recently, Shin et al*.* developed an HDL-mimetic formulation that effectively displays multivalent ApoA-I peptides on the surfaces of protein nanotubes derived from TMV and virus-like particles sourced from bacteriophage Qβ. This formulation significantly enhances the efficiency of cholesterol efflux [207]. Mu et al*.* employed hyaluronic acid (HA)-coated poly(ethylene glycol)-poly(tyrosine ethyl oxalyl) (PEG-Ptyr-EO) biodegradable polymeric micelles for the encapsulation of simvastatin (SIM) [208]. The outer layer of HA specifically targeted inflammatory macrophages (CD44-positive), while the inner PEG-Ptyr-EO material was not only enzymatically degradable but also exhibited ROS responsiveness. This dual functionality allowed it to effectively consume ROS at the pathological site, thereby inhibiting the accumulation of proinflammatory macrophages and alleviating oxidative stress. Experiments demonstrated that this system could be effectively internalized by inflammatory cells and exhibits high cytotoxicity, while showing low toxicity to normal cells. In animal models, this system significantly reduced cholesterol levels in atherosclerotic plaques, exerting notable therapeutic effects. In another case, Xie et al*.* developed rod-shaped tubular micromotors by utilizing electrospun fiber segments as templates for the synthesis of polydopamine (PDA) microtubes [209]. These microtubes were loaded with urokinase plasminogen activator (uPA) and surface-modified with fucoidan (Fu), while simultaneously incorporating Escherichia coli Nissle 1917 (EcN) to develop hybrid micromotors. This approach not only extended the half-life of uPA and improved its bioavailability but also significantly enhanced thrombolytic capability, providing a promising therapeutic strategy for atherosclerosis.

### MI

**Fig. 16:**
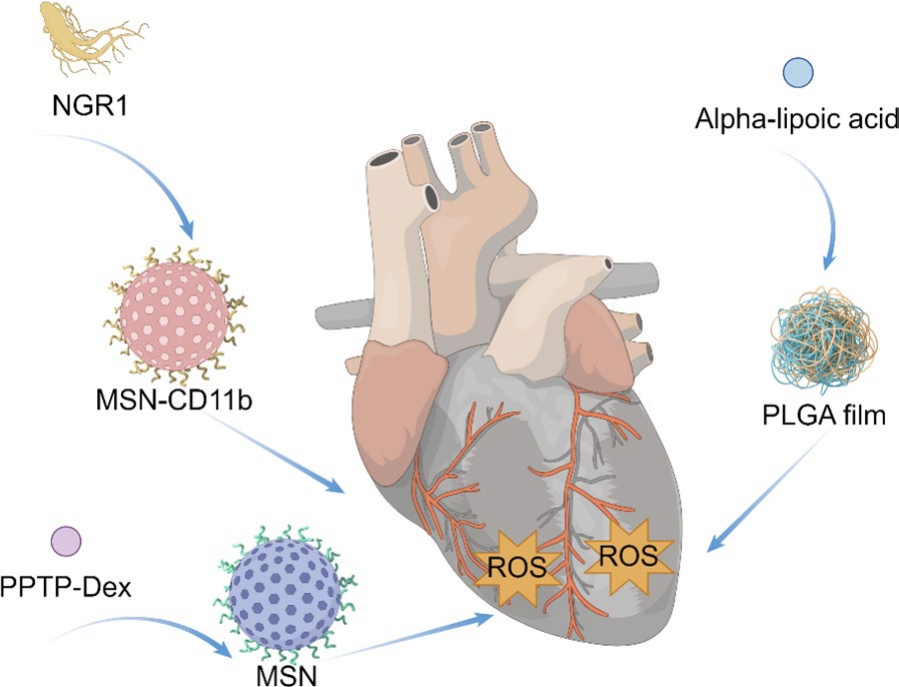
Nano-formulations for the therapy of MI

Clinically, growth factors, cytokines, and small molecule compounds are predominantly utilized for the treatment of myocardial ischemia. The ischemic regions of the myocardium demonstrate elevated vascular permeability, facilitating targeted delivery through the passive accumulation of nanodrug carriers. Additionally, the ischemic areas of the myocardium are frequently infiltrated by monocytes and macrophages, which can actively internalize nano-formulations for drug delivery via phagocytosis. Currently, nano-formulations have demonstrated revolutionary potential in the field of myocardial infarction treatment, with their precise drug delivery and efficient biological effects paving the way for novel approaches in myocardial protection and repair (Fig. 16).

Oxidative stress, characterized by excessive accumulation of reactive oxygen species (ROS), is associated with acute myocardial infarction (AMI) and the injury resulting from vascular reperfusion therapy. α-lipoic acid (α-LA) is a potent antioxidant; however, its therapeutic application is constrained by its rapid clearance and extensive distribution. Xie et al*.* utilized PLGA copolymer as a carrier to fabricate an electrospun LA@PLGA film, enabling the controlled release of LA to mitigate ROS-induced damage following myocardial injury and restore cardiac function [210]. Notoginsenoside R1 (NGR1), an active compound derived from Panax notoginseng, demonstrates therapeutic potential in the treatment of MI. Han et al*.* designed mesoporous silica NPs loaded with NGR1 and conjugated with CD11b antibodies (MSN-NGR1-CD11b), which are capable of specifically recognizing monocytes and neutrophils at the infarction site. In MI mice, intravenous administration of these NPs enhances cardiac function, stimulates angiogenesis, decreases apoptosis, and modulates macrophage polarization along with inflammatory cytokines and chemokines [211]. The receptor for advanced glycation endproducts (RAGE) has been implicated in a variety of chronic inflammatory conditions.

Lan et al*.* developed a ROS-degradable polycation, PPTP, specifically targeting myocardial inflammation resulting from ischemia/reperfusion (I/R) injury [212]. PPTP was utilized to functionalize mesoporous silica NPs (MSN) encapsulating dexamethasone (Dex), while simultaneously condensing RAGE small interfering RNA (siRAGE) and serving as a gatekeeper for MSN to prevent premature leakage of Dex. In a rat model of IR injury, the nanotherapeutic was efficiently delivered to inflamed cardiomyocytes via PGE2-mediated transport. The excessive ROS generated during IR injury degraded PPTP, leading to the controlled release of siRAGE and Dex, thereby achieving effective silencing of RAGE and a synergistic anti-inflammatory effect.

### Other cardiovascular diseases

Significant advancements have been achieved in the research on nano-formulations for the treatment of various cardiovascular diseases, including hypertension, pulmonary arterial hypertension (PAH), and ischemic stroke. Currently, a wide range of antihypertensive drugs are available in clinical practice; however, these medications exhibit certain limitations, including low bioavailability and a short plasma half-life. In contrast, nano-formulations offer substantial advantages in these aspects. Telmisartan is an orally active, non-peptide angiotensin II receptor antagonist with relatively low water solubility, commonly utilized in the management of hypertension. Nevertheless, the utilization of telmisartan nanocrystals can significantly enhance oral bioavailability [213]. The curcumin nano-emulsion developed by Rachmawati et al*.* demonstrated a modest enhancement in its inhibitory effect on angiotensin-converting enzyme (ACE) [214]. Furthermore, regarding cholesterol reduction, the curcumin nano-emulsion demonstrates substantial efficacy, even outperforming the standard drug pravastatin.

PAH is a severe cardiopulmonary disorder characterized by the excessive proliferation and inflammation of pulmonary artery smooth muscle cells (PASMCs), for which effective therapeutic options remain limited [215, 216]. Teng et al*.* co-delivered the apoptosis-executing gene p53 and the anti-inflammatory baicalein to PASMCs via a carrier-free method, with the objective of alleviating PAH [217]. The in vivo experiments demonstrated that this system specifically targets the lung-pulmonary artery-PASMC axis, thereby reducing pulmonary artery pressure, downregulating the pro-inflammatory cytokine TNF-α, and inhibiting pulmonary artery and right ventricular remodeling. Consequently, it effectively reverses monocrotaline-induced PAH. In another case, Vani et al*.* demonstrated that multi-hydroxy fullerene derivatives substantially reduced the area of brain injury in rats subjected to ischemic stroke, and significantly lowered the levels of malondialdehyde (MDA) and nitrate in the ischemic hemisphere [218]. Furthermore, the administration of fullerene NPs resulted in a significant increase in glutathione (GSH) content and superoxide dismutase (SOD) activity. This enhancement effectively mitigated the elevated levels of free radicals under ischemic conditions, thereby providing robust protection against ischemic/reperfusion injury to brain cells.

**Fig. 17:**
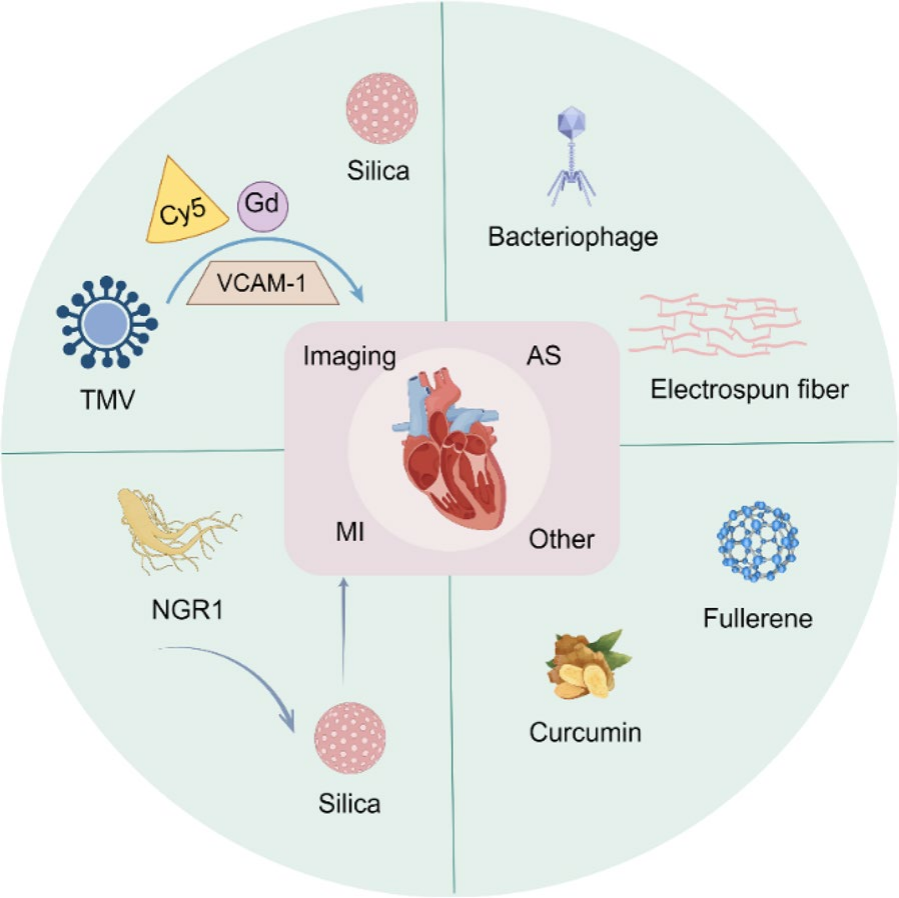
Nano-formulations in the therapy for cardiovascular diseases

It can thus be seen that nano-formulations will one day also become a powerful tool for the diagnosis and treatment of cardiovascular diseases (Fig. 17).

## Nano-formulation applications in treating diabetes

**Fig. 18:**
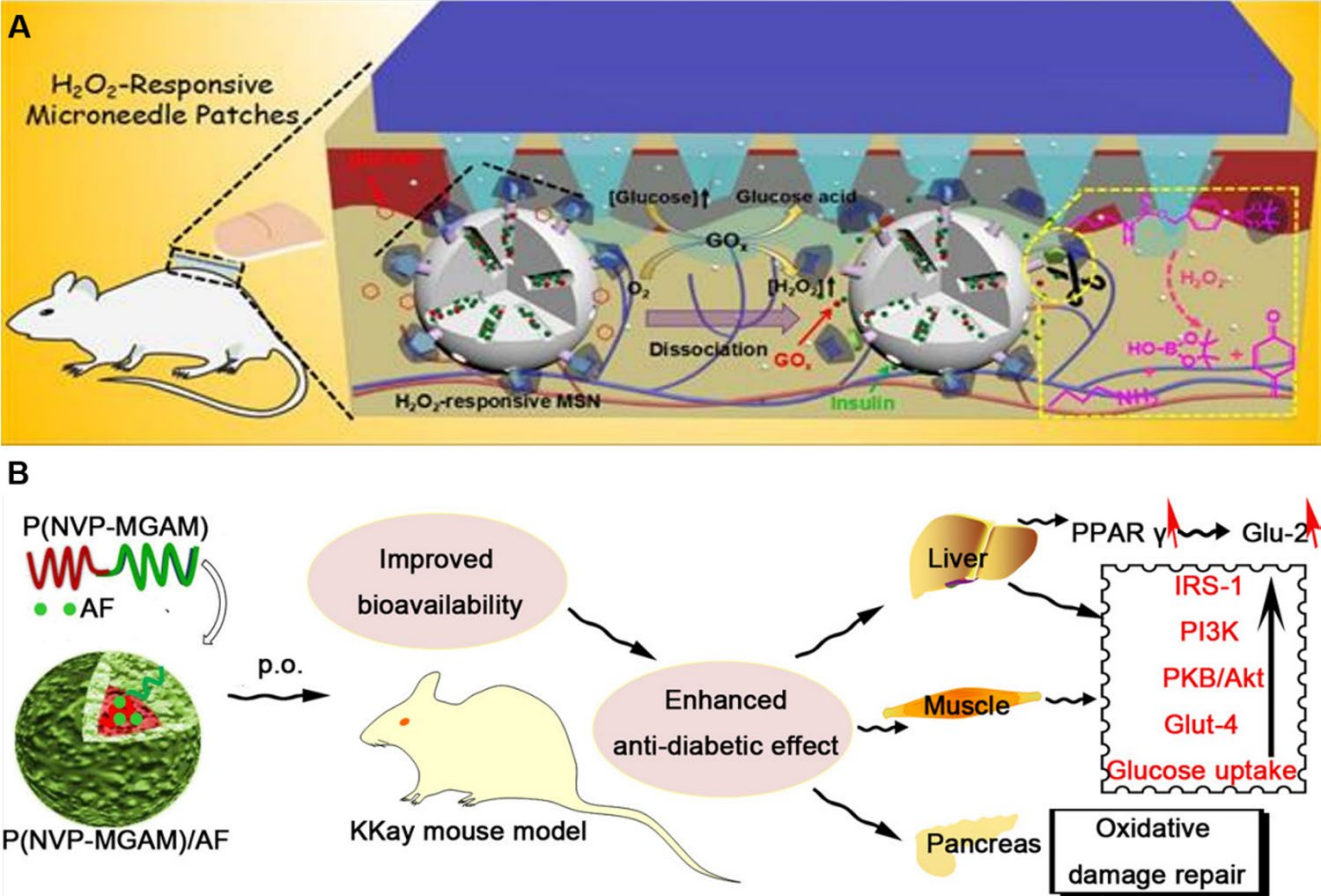
Schematic model of nano-formulations in treating diabetes. **A** Schematic illustration of the action of insulin-loaded and H_2_O_2_-responsive mesoporous silica nanoparticles (MSNs). **B** Schematic illustration of the action of Amentoflavone-loaded P (NVP-MGAM)/AF oral microspheres *Adapted from Xu B, Jiang G, Yu W, *et al*. H*_*2*_*O*_*2*_*-Responsive mesoporous silica nanoparticles integrated with microneedle patches for the glucose-monitored transdermal delivery of insulin. J Mater Chem B. 2017;5(41):8200–8. Zhang J, Zhou J, Zhang T, *et al*. Facile Fabrication of an Amentoflavone-Loaded Micelle System for Oral Delivery To Improve Bioavailability and Hypoglycemic Effects in KKAy Mice. ACS Applied Materials & Interfaces. 2019;11(13):12,904–13*

Diabetes has emerged as one of the prevalent metabolic diseases globally, characterized by inadequate insulin secretion and/or action, leading to hyperglycemia [219, 220]. Anti-diabetic nano-formulations represent a significant advancement in the field of diabetes treatment, capable of reducing enzymatic degradation of specific anti-diabetic drugs, such as insulin, within the gastrointestinal (GI) tract. This enhancement improves drug delivery efficiency and therapeutic efficacy [221, 222]. Nano-formulations exhibit multifaceted application potential in diabetes therapy, as illustrated in Fig. 18.

Hunt et al*.* have developed a novel oral insulin nano-formulation consisting of insulin-conjugated silver sulfide quantum dots coated with chitosan/glucose polymers [223]. This formulation remains insoluble in acidic environments but demonstrates significantly enhanced absorption at neutral pH levels, such as those observed in the human duodenum. Additionally, it is sensitive to glucosidase, which triggers insulin release. Experiments have demonstrated that this formulation significantly reduces blood glucose levels in mice, rats, and non-diabetic baboons, with no observed adverse effects such as hypoglycemia, weight gain, or hematological toxicity. Xu et al*.* have designed a novel microneedle (MN) delivery device integrated with insulin-loaded and H_2_O_2_-responsive MSNs [224]. These MSNs, synthesized via targeted modification and host–guest complexation, exhibit a glucose-responsive behavior. Upon the conversion of glucose to gluconic acid and the concurrent generation of hydrogen peroxide, the chemical bonds on the MSN surface undergo oxidative cleavage, resulting in NP disintegration and subsequent insulin release. In experiments conducted on diabetic rats, this device exhibited significant hypoglycemic efficacy following percutaneous administration, as compared to subcutaneous injection.

Shrestha et al*.* have developed a nanosystem capable of simultaneously delivering glucagon-like peptide-1 (GLP-1) and dipeptidyl peptidase-4 (DPP4) inhibitors [225]. The system, which employs chitosan-modified porous silicon nanoparticles as the core, simultaneously encapsulates GLP-1 and DPP4 inhibitors to achieve a synergistic effect, thereby enhancing insulin secretion and inhibiting GLP-1 degradation. The intestinal polymer coating serves as a protective barrier against acid-induced and enzymatic degradation, thereby safeguarding the drugs from the adverse effects of the harsh gastrointestinal environment. Oral administration of this nano-system significantly reduced blood glucose levels and increases pancreatic insulin content. Polyphenols, as secondary metabolites in plants, exhibit potential antidiabetic properties [226]. Zhang et al*.* pioneered the development of the P(NVP-MGAM)/AF oral micellar system, which markedly enhanced the oral bioavailability of hop flavonoids [227]. In a mouse model of diabetes characterized by insulin resistance, this micellar system exhibited significant therapeutic efficacy via mechanisms including the regulation of blood lipids, attenuation of inflammation, and activation of PPARγ as well as the PI3K/Akt signaling pathways.

## Opportunities for nano-formulations in treating skin disease

Skin conditions represent one of the most widespread health concerns, imposing considerable economic and psychological burdens on affected individuals. Owing to the barrier function of the skin and the unfavorable physicochemical properties of certain drugs, transdermal drug delivery poses a considerable challenge [228]. Skin conditions can primarily be categorized into two groups: barrier-impaired skin conditions, which include atopic dermatitis, psoriasis, and fungal infections; and follicular disorders, which encompass acne and alopecia [229]. Barrier-impaired skin diseases predominantly affect the active epidermal cell layer and the dermis, whereas targeting hair follicles is essential for the effective treatment of follicular skin conditions. Advancements and innovations in drug delivery systems, especially with the emergence of nano-formulations, have facilitated significant breakthroughs in the treatment of dermatological conditions [230].

Tacrolimus (TRL), an immunosuppressive agent utilized in the management of atopic dermatitis (AD), demonstrates efficacy; however, its clinical application is constrained by its poor water solubility, low permeability, and potential cytotoxicity. To address these challenges, Ren et al*.* innovatively developed a novel formulation by embedding tacrolimus-loaded nano-transfersomes (TRL-NTs) into chitosan gel, thereby forming the TRL-NTs gel (TRL-NTsG) [231]. TRL-NTsG not only demonstrates markedly superior efficiency in the release of tacrolimus compared to traditional formulations, but also achieves substantial improvements in permeability and stability. In an animal model of AD, mice treated with TRL-NTsG exhibited a significant reduction in ear thickness. Furthermore, these mice showed a notable decrease in IgE levels, suggesting that TRL-NTsG may effectively mitigate AD-associated immune responses. In another study, Fratoddi et al*.* modified AuNPs using 3-mercapto-1-propanesulfonic acid salt, resulting in AuNPs-3MPS, which were subsequently loaded with the immunosuppressant methotrexate (MTX) [232]. AUNPS-3MP confers novel chemical and physical properties on AuNPs, such as an enhanced drug-loading capacity, improved stability, and increased targeting ability. The AUNPS-3MP coating effectively mitigates the degradation of MTX during delivery, thereby preserving its immunosuppressive activity. AuNPs inherently exhibit excellent biocompatibility and degradability. Furthermore, after modification with AUNPS-3MP, their immunogenicity is significantly diminished, making them highly suitable for long-term administration. The resultant AuNPs-3MPS@MTX formulation significantly mitigated scales, erythema, epidermal thickness, and inflammatory infiltration in a murine model of psoriasis. Furthermore, the solid lipid NPs loaded with fluconazole (FLZ-SLN) developed by Moazeni et al*.* have also shown efficacy against strains exhibiting reduced sensitivity to conventional fluconazole formulations [233]. The alterations in the microenvironment of hair follicles caused by acne, including an elevation in pH levels, have prompted innovative therapeutic approaches.

**Fig. 19:**
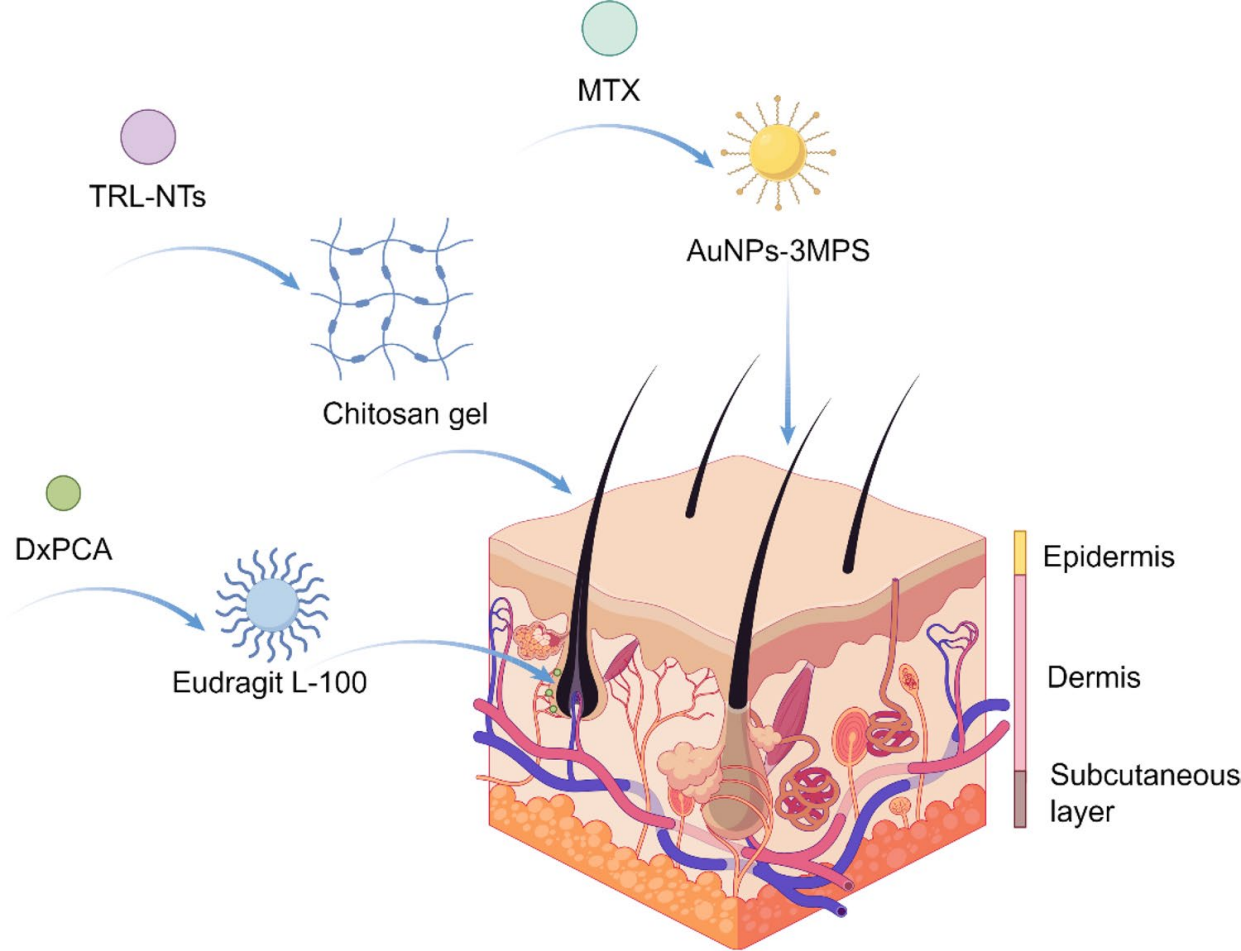
Nano-formulations in treating skin disease

Dong et al*.* developed pH-sensitive polyacrylic resin I (Eudragit^®^ L-100) NPs encapsulating spin-labeled dexamethasone (DxPCA) [234]. The findings demonstrated that DxPCA-Eudragit^®^ L-100 NPs exhibited markedly enhanced skin penetration and drug release properties, facilitating targeted delivery to hair follicles for the treatment of dermatological conditions such as acne. Minoxidil serves as an efficacious topical therapy for hair loss; however, it is constrained by a slow onset of action and limited efficacy. Moreover, it lacks the capability to effectively mitigate excessive oxidative stress, a significant pathogenic factor contributing to hair loss. Xiao et al*.* leveraged the transition metal molybdenum, known for its rapid electron transfer properties, to mitigate oxidative stress [235]. They conducted an intervention study on a mouse hair growth model using nano-molybdenum, minoxidil, and a combination of both treatments. The findings indicated that nano-molybdenum not only accelerated hair growth and increased the number of hair follicles but also reduced the expression levels of molecules associated with oxidative stress. Furthermore, when combined with minoxidil, nano-molybdenum exhibited a synergistic effect in promoting hair growth. In summary, nano-formulations have demonstrated immense potential and a broad application prospect in the field of skin disease treatment (Fig. 19).

## Potential role of nano-formulations in treating eye diseases

**Fig. 20:**
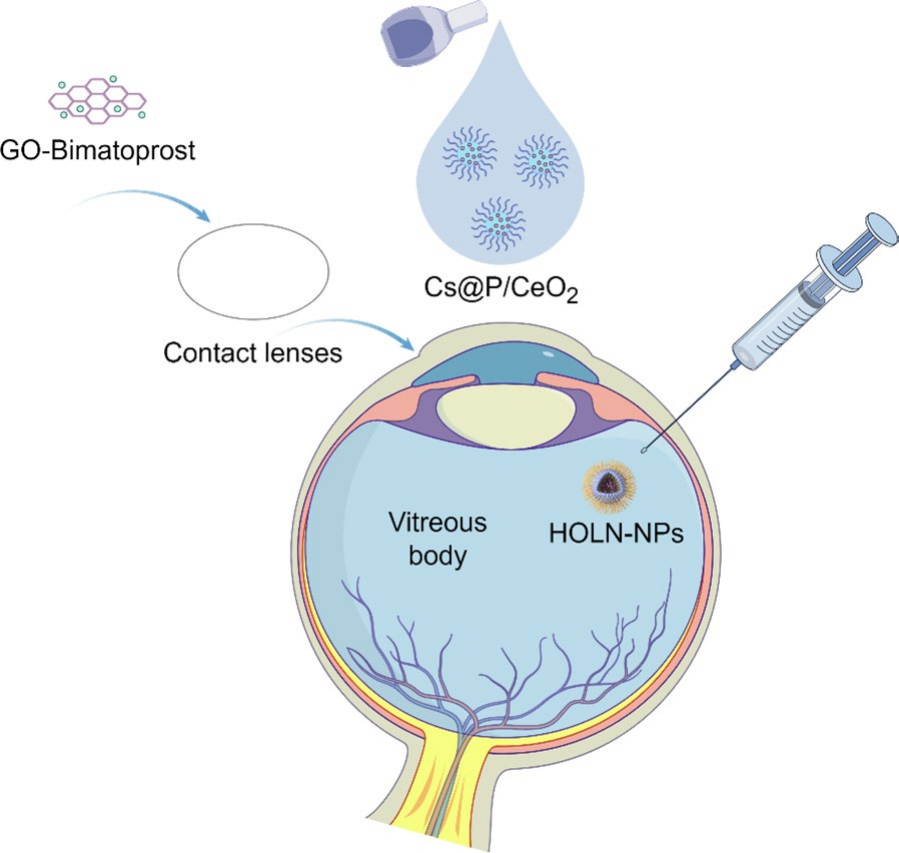
Nano-formulations in treating ocular diseases

Eye diseases exert a significant impact on both the visual function and overall quality of life for patients globally [236]. Nevertheless, the unique and intricate anatomical and physiological features of the eye present substantial challenges for achieving effective ocular drug delivery within the realms of clinical pharmacology and biomaterials science [237]. To address these challenges, nano-formulations for ophthalmic use with precise delivery characteristics have emerged [238]. Researchers have developed a range of innovative nanotechnology-based drug delivery systems designed to extend drug residence time in the eye and enhance corneal permeability, thereby improving the bioavailability of drugs within ocular tissues (Fig. 20).

Dry eye disease (DED) is progressively impacting an increasingly large global population, with its etiology encompassing ocular surface inflammation and oxidative stress [239]. Cui et al*.* have developed a nanoceramic material loaded with cyclosporine A (Cs@P/CeO_2_), which exhibits prolonged antioxidant and anti-inflammatory properties due to its regenerative antioxidant activity and sustained release of cyclosporine A [240]. Their findings indicated that Cs@P/CeO_2_ could restore the balance of immune-epithelial communication in the corneal microenvironment, reduce the polarization of inflammatory macrophages, and mitigate oxidative stress. Nano-ceria exhibits self-regenerative antioxidant capacity, which stems from the reversible switching between Ce^3^⁺ and Ce^4^⁺ ions due to oxygen vacancies. Simultaneously, it is loaded with the immunomodulatory agent Cyclosporin A, thereby achieving dual functionalities of antioxidant defense and immunomodulatory effects. The eye drops are modified with PEG to enhance the biocompatibility and hydrophilicity of nano-ceria, and to enable slow-release of cyclosporine A, thereby prolonging the ocular surface residence time and providing long-term antioxidant and anti-inflammatory effects.

Glaucoma is a progressive and irreversible ocular disorder characterized by the degeneration and loss of retinal ganglion cells (RGCs) [241]. Zhou et al*.* have developed a dual-drug conjugate (OLN monomer) that is responsive to ROS [242]. This conjugate, which integrates nicotinamide and oleic acid, can self-assemble into NPs (uhOLN-NPs) with ROS scavenging capabilities. Subsequently, the researchers encapsulated these uhOLN-NPs using a hypoxia-responsive polymer, resulting in NPs (HOLN-NPs) that exhibit responsiveness to both hypoxia and ROS. When RGCs are subjected to hypoxic conditions or elevated ROS levels, HOLN-NPs release uhOLN-NPs, which subsequently release nicotinamide and oleic acid. These compounds not only effectively scavenge ROS but also activate the CaMKII/CREB signaling pathway, thereby providing protection for mitochondrial function in RGCs. The utilization of contact lenses as a vehicle for ocular drug delivery is regarded as a promising alternative to traditional eyedrops. However, this approach encounters challenges including suboptimal drug uptake efficiency, lens hydration-induced swelling, and diminished optical transmittance [243].

2D materials can also be utilized for treating eye diseases. For instance, Maulvi et al*.* incorporated graphene oxide (GO) into silicone hydrogel contact lenses [244]. These lenses were either immersed in a bimatoprost solution or fabricated with bimatoprost directly incorporated during the lens polymerization process, resulting in contact lenses with or without GO. The incorporation of GO into the contact lenses effectively regulated the release of bimatoprost, while enhancing the lens's swelling behavior and optical transmittance properties. Compared to the administration of eyedrops, the use of contact lenses containing GO and an appropriate concentration of bimatoprost markedly increased the mean residence time (MRT) and area under the curve (AUC) of the drug.

## Challenges and prospects

Nano-formulations, resulting from the integration of cutting-edge technology and medical expertise, are quietly spearheading a transformative shift in the medical field. They function as precise “drug delivery vehicles”, capable of directly administering therapeutic agents to specific cells or tissues within the human body. This approach not only significantly enhances drug efficacy but also markedly reduces adverse effects, thereby realizing the true potential of targeted therapy. In the field of drug delivery, nano-formulations exhibit unparalleled advantages. They are capable of efficiently traversing biological barriers and accurately delivering drugs to the site of lesions, be it malignant tumors, chronic pulmonary conditions, complex cardiovascular diseases, or refractory conditions such as diabetes, fragile ocular diseases, and recalcitrant dermatological disorders, thereby enabling more precise and effective therapeutic interventions. Furthermore, nano-formulations are capable of achieving stable drug release within the body, thereby maintaining therapeutic concentrations and prolonging treatment duration, which ultimately enhances therapeutic efficacy. In the realm of diagnostics and imaging, the utilization of nanoprobes is equally significant. They can interact with specific cells or molecules, thereby providing more precise and sensitive information for disease diagnosis. Leveraging advanced imaging technologies, physicians can clearly visualize changes at the lesion site, offering robust support for early detection and treatment of diseases.

Nevertheless, the application of nano-formulations faces several challenges. Potential toxicity and biocompatibility concerns have consistently posed significant challenges that impede their advancement. Ensuring the safety and stability of nano-formulations within biological systems while preventing potential damage to healthy tissues remains a critical challenge that researchers must urgently address. Furthermore, the limitations encountered during the delivery process of NPs pose significant challenges to their clinical translation. Currently, there is a significant gap in our comprehensive understanding of the disposition and metabolic pathways of nanodrugs within the human body, thereby heightening the risks and uncertainties inherent in drug development. Regarding quality control, nano-formulations also encounter substantial challenges. Parameters such as diameter, size distribution, morphology, surface charge, drug loading capacity, and release profile are directly correlated with the stability and efficacy of nano-formulations. Consequently, the establishment of a more rigorous and precise quality control system to ensure that each batch of nano-formulations achieves optimal therapeutic outcomes represents a significant challenge that researchers must address.

Despite encountering numerous challenges, the prospects for the development of nano-formulations remain promising. It is imperative to conduct in-depth research into critical areas such as the mechanisms of toxicity associated with nano-formulations, strategies to enhance biocompatibility, optimization methods for delivery systems, and advancements in quality control technologies. Currently, advanced materials, coatings, or surface modifications are being developed to enhance biocompatibility. For instance, biodegradable materials including PLGA, chitosan, liposome and other detachable carrier (e.g. hydrolysis of silicon-based nanoparticles to silicic acid), have be1en used to reduce long-term toxicity. Surface modification using by Pegs (pegylation) or coating with hydrophilic polymers (e.g., polyvinylpyrrolidone, PVP) can reduce immunogenicity and avoid clearance of the RES [245]. pH, enzyme, or REDOX responsive nano-formulations can be designed to release drugs in specific microenvironments (such as low pH or high glutathione environments). Furthermore, surface engineering strategies, including charge regulation and bionic design utilizing exosomes or lipoprotein-mimic nanoparticles, have been developed to enhance biocompatibility. The "optical switch nanocarriers" developed by Stanford University enable precise control over the timing of release through varying wavelengths of light [246]. Additionally, selenide nanoparticles have emerged as a research focus owing to their enhanced redox sensitivity. In addition, AI facilitates the acceleration of nanomaterial screening, drug carrier optimization, and clinical trial design by enhancing computational efficiency and data analysis capabilities. Generative AI models, such as Google DeepMind's Material Exploration Network, predict thousands of stable nanostructures. AI-designed peptide nanotubes have the ability to self-assemble into scaffolds for drug delivery (for Alzheimer's disease) [247]. Optimization of drug delivery using AI technology especially the machine learning can predict release dynamics. The MIT team employed neural networks to optimize the polymer composition of nanoparticles, thereby reducing the drug release time error to less than 5% [248]. NVIDIA's Clara platform simulates the distribution of nano-formulation within the human body, thereby minimizing the reliance on animal testing. Deep learning integrated with AI-based screening for targeted ligands is employed to analyze tumor cell surface proteome data and design specific peptide sequences, such as nanoparticles targeting PD-L1. We look forward to the emergence of more innovative nano-formulations that will not only improve treatment outcomes but also enhance the quality of life for patients.

## Conclusions

By precisely controlling drug delivery and release, nano-formulations not only significantly improve therapeutic effects but also reduce the side effects associated with traditional treatments, offering new possibilities for the treatment of multiple diseases. Although challenges such as toxicity, biocompatibility, delivery efficiency, and quality control arise in practical applications, these issues are gradually being addressed through continuous scientific research and technological advancements. In the future, nano-formulations are expected to play a pivotal role in the treatment of more diseases, further enhancing medical standards, improving patients' quality of life, and bringing revolutionary changes to the field of medicine.

In summary, we presented a comprehensive review on the design and synthesis of nano-formulations, action mechanisms, applications in molecular imaging, and the treatment of various diseases. In particular, we systematically elucidate the application of novel nano-formulations in disease diagnosis and treatment, while critically analyzing the difficulties and challenges encountered during implementation. Despite being rigorously validated through extensive research, such detailed and comprehensive studies remain underreported in the existing literature.

## Data Availability

No datasets were generated or analysed during the current study.

## Abbreviations

MBC: Metastatic breast cancer
PC: Pancreatic cancer
NSCLC: Non-small cell lung cancer
OC: Ovarian cancer
T-AML: Therapy-related acute myeloid leukemia
AML-MRC: AML with myelodysplasia-related changes
siRNA: Small interfering RNA
aTTP: Acquired thrombotic thrombocytopenic purpura
mRNA: Messenger RNA
NPs: Nanoparticles
PLA: Polylactic acid
PGA: Polyglycolic acid
NK: Natural killer
BNPs: Biomimetic nanoparticles
EPR: Enhanced permeability and retention
NDs: Nanodiscs
HDLs: High-density lipoproteins
SR-B1: Scavenger Receptor Class B Type 1
FARs: Folate receptors
LDL: Low-density lipoprotein
FI: Fluorescence imaging
MRI: Magnetic resonance imaging
CT: Computed tomography
PET: Positron emission tomography
PEG-LIP: Pegylated liposomes
RGD: Arg-Gly-Asp
NIR: Near-infrared
MNPs: Magnetic nanoparticles
ROS: Reactive oxygen species
CCM-NPs: Cancer cell membrane-coated nanoparticles
ICG: Indocyanine green
FL/PA: Fluorescence/photoacoustic
EMT: Epithelial-to-Mesenchymal Transition
WFA: Withaferin-A
AuNPs: Gold nanoparticles
ABCG2: ATP-binding cassette subfamily G member 2
DNC: Dendritic nanoparticles
PD-L1: Programmed cell death ligand 1
DTX: Docetaxel
BCM: Borate cross-linked micelle
PTX: Paclitaxel
NCM: Non-cross-linked micelles
HPMAm: Hydroxypropyl methacrylamide
HSP90: Heat shock protein 90
RES: Reticuloendothelial system
ACT: Adoptive cell therapy
MAB: Monoclonal antibody
PFS: Progression-free survival
OS: Overall survival
ICIs: Immune checkpoint inhibitors
PD-1: Programmed cell death protein 1
HL: Hodgkin lymphoma
CTLA-4: Cytotoxic T-lymphocyte-associated protein 4
CRC: Colorectal cancer
PROTACs: Protein hydrolysis-targeting chimeras
POI: Protein of interest
PSRNs: PH/cathepsin B sequentially responsive nanoparticles
CDK4/6: Cyclin-dependent kinases 4 and 6
CRC: Colorectal cancer
ICBs: Immune checkpoint blockades
CAR-T: Chimeric antigen receptor T cell immunotherapy
CARs: Chimeric antigen receptors
NGs: Nanogels
TCR: T-cell receptor
EGFR: Epidermal growth factor receptor
PSMA: Prostate-specific membrane antigen
HER2: Human epidermal growth factor receptor 2
ADCs: Antibody–drug conjugates
Tmab: Trastuzumab
APCs: Antigen-presenting cells
DC: Dendritic cell
αPD-L1: Anti-PD-L1 antibody
CTLs: Cytotoxic T lymphocytes
LNPs: Lipid nanoparticles
TME: Tumor microenvironment
TRK: Tropomyosin receptor kinase
TAMs: Tumor-associated macrophages
OMVs: Outer membrane vesicles
mRNA: Messenger RNA
PBA: Phenylboronic acid
SA: Sialic acid
DOX: Doxorubicin
MEs: Milk-derived exosomes
PDT: Photodynamic Therapy
PTT: Photothermal Therapy
AuNRs: Gold nanorods
PLK1: Polo-like kinase-1
MDR: Multidrug resistance
ABC: ATP-binding cassette
COPD: Chronic obstructive pulmonary disease
CVD: Cardiovascular diseases
MI: Myocardial infarction
CT-PET: Computed tomography-positron emission tomography
USPIO: Ultra-small superparamagnetic iron oxide
TMV: Tobacco mosaic virus
TAP: Thrombin-activated fluorescent peptides
SPIONs: Superparamagnetic iron oxide nanoparticles
AS: Atherosclerosis
VSMCs: Vascular smooth muscle cells
TF: Tissue factor
HA: Hyaluronic acid
SIM: Simvastatin
PDA: Polydopamine
uPA: Urokinase plasminogen activator
Fu: Fucoidan
AMI: Acute myocardial infarction
α-LA: α-Lipoic acid
NGR1: Notoginsenoside R1
RAGE: Receptor for advanced glycation endproducts
I/R: Ischemia/reperfusion
MSN: Mesoporous silica nanoparticles
Dex: Dexamethasone
PAH: Pulmonary arterial hypertension
ACE: Angiotensin-converting enzyme
PASMCs: Pulmonary artery smooth muscle cells
MDA: Malondialdehyde
GSH: Glutathione
SOD: Superoxide dismutase
GI: Gastrointestinal
MN: Microneedle
GLP-1: Glucagon-like peptide-1
DPP4: Dipeptidyl peptidase-4
TRL: Tacrolimus
AD: Atopic dermatitis
MTX: Methotrexate
DED: Dry eye disease
RGCs: Retinal ganglion cells
GO: Graphene oxide
MRT: Mean residence time
AUC: Area under the curve
